# Enhanced Fluorescent Siderophore Biosynthesis and Loss of Phenazine-1-Carboxamide in Phenotypic Variant of *Pseudomonas chlororaphis* HT66

**DOI:** 10.3389/fmicb.2018.00759

**Published:** 2018-04-23

**Authors:** Yang Liu, Zheng Wang, Muhammad Bilal, Hongbo Hu, Wei Wang, Xianqing Huang, Huasong Peng, Xuehong Zhang

**Affiliations:** ^1^State Key Laboratory of Microbial Metabolism, School of Life Sciences and Biotechnology, Shanghai Jiao Tong University, Shanghai, China; ^2^National Experimental Teaching Center for Life Sciences and Biotechnology, Shanghai Jiao Tong University, Shanghai, China

**Keywords:** *Pseudomonas chlororaphis*, spontaneous phenotypic variant, phenazine-1-carboxamide, siderophore biosynthesis, pyoverdine, GacS, RsmX/Y/Z

## Abstract

*Pseudomonas chlororaphis* HT66 is a plant-beneficial bacterium that exhibits wider antagonistic spectrum against a variety of plant pathogenic fungi due to its main secondary metabolite, i.e., phenazine-1-carboxamide (PCN). In the present study, a spontaneous phenotypic variant designated as HT66-FLUO was isolated from the fermentation process of wild-type HT66 strain. The newly isolated phenotypic variant was morphologically distinct from the wild-type strain such as larger cell size, semi-transparent, non-production of PCN (Green or yellow crystals) and enhanced fluorescence under UV light. The whole-genome, RNA-sequencing, and phenotypic assays were performed to identify the reason of phenotypic variation in HT66-FLUO as compared to the HT66. Transcriptomic analysis revealed that 1,418 genes, representing approximately 22% of the 6393 open reading frames (ORFs) had undergone substantial reprogramming of gene expression in the HT66-FLUO. The whole-genome sequence indicated no gene alteration in HT66-FLUO as compared to HT66 according to the known reference sequence. The levels of global regulatory factor *gacA* and *gacS* expression were not significantly different between HT66 and HT66-FLUO. It was observed that overexpressing *gacS* rather than *gacA* in HT66-FLUO can recover switching of the variant to HT66. The β-galactosidase (*LacZ*) activity and qRT-PCR results indicate the downregulated expression of *rsmX, rsmY*, and *rsmZ* in HT66-FLUO as compared to HT66. Overexpressing three small RNAs in HT66-FLUO can revert switching of colony phenotype toward wild-type HT66 up to a certain degree, restore partial PCN production and reduces the fluorescent siderophores yield. However, the origin of the spontaneous phenotypic variant was difficult to be determined. In conclusion, this study helps to understand the gene regulatory effect in the spontaneous phenotypic variant.

## Introduction

Phenazines are nitrogen-containing heterocyclic secondary metabolites produced by Gram-negative and Gram-positive strains such as *Pseudomonas* spp. and *Streptomyces* spp., respectively. Owing to their virulence-related roles and broader antibiotic spectrum, these nitrogen-containing compounds function as biocontrol agents against a variety of plant pathogens (Mazzola et al., 1992; Laursen and Nielsen, 2004). Therefore, phenazine and its derivatives have used for diverse applications including electron shuttling, environmental sensors and biosensors, and central components of antitumor compounds (Pierson and Pierson, 2010). Phenazines have considerable potentialities to modify cellular redox states, act as cell signals that regulate patterns of gene expression, contribute to biofilm formation and architecture, and enhance bacterial survival. They also effect on eukaryotic hosts and host tissues, including the modification of multiple host cellular responses (Van Wees et al., 2008).

Siderophores (iron carriers) are low-molecular-weight iron (Fe^3+^) chelating molecules produced by bacteria under iron-limiting conditions (Andrews et al., 2003; Braun and Hantke, 2011). Microorganisms produce one primary high-affinity siderophore and one or several lower-affinity siderophores to acclimatize iron fluctuating conditions. An accurate expression of siderophores can help bacteria to maintain intracellular iron homeostasis and enhances their environmental adaptability (Yu et al., 2014). Pyoverdines are water-soluble fluorescent pigments of the fluorescent *Pseudomonas* species. It is also considered as a powerful Fe^3+^ scavenger, efficient Fe^3+^ transporter, and essential for bacterial survival, such as biofilm formation and competitiveness in *P. aeruginosa* (Handfield et al., 2000). In the plant pathogen such as *P. syringae*, pyoverdines were proved to be important colonization factors, and necessary to establish a link between quorum sensing, iron uptake and virulence behavior (Taguchi et al., 2010). Various secondary siderophores such as pyochelin, achromobactin, thioquinolobactin, pseudomonin, and yersiniabactin, are produced by bacteria in iron-deficient conditions to store energy (Ravel and Cornelis, 2003; Yu et al., 2014).

Also, bacteria enhance their adaptability and biological diversity under certain conditions using a phenotypic or phase variation. Phenotypic or phase variation is mediated by DNA mutation and reorganization or modification (van den Broek et al., 2005a). Phase variation appears at a high frequency in every generation and can bring multiple phenotypic modulations and on-off switching of physiological features (van den Broek et al., 2005a). Although, phase variation or antigenic variation has primarily been associated with host–pathogen interactions, however, several reports describe phase and phenotypic variations in a broader context. Such phenotypic variations are involved in the production of exo-enzymes and other secondary metabolites affecting colonization behavior and biocontrol activity of rhizosphere bacteria (van den Broek et al., 2003). In *Pseudomonas*, a small-colony variant (SCVs) is the most common type of naturally occurring phenotypic variation. Many *Pseudomonas* strains undergo phenotypic diversification while adapting to the biofilm environment. SCVs are generally correlated with smaller colony sizes and increase resistance to antibiotics. Isolated SCVs are often unstable and can rapidly switch back to the wild-type phenotype (Wang et al., 2015). The appearance of SCVs is related to the multiple selective pressures and a diverse genetic basis. Another frequently occurring phenotypic variation is a spontaneous mutant in GacS/GacA two-component regulatory system. The GacS/GacA composed of the sensor kinase GacS and its cognate response regulator, GacA, is highly conserved in *Pseudomonas*. The GacS/GacA system initiate the Gac-Rsm cascade and then activates the transcription of three sRNAs (RsmX/Y/Z), which subsequently sequester the small RNA-binding proteins RsmA and RsmE. These proteins prevent ribosome binding at the mRNA of target genes to relieve translational repression (Blumer et al., 1999; Humair et al., 2010; Duss et al., 2014). Phenotypic variants with *gacS* or *gacA* mutations have obvious traits, for example, reduced secondary metabolites (phenazine, quorum-sensing signals, exo-proteases) production and enhanced siderophores yield (Poritsanos et al., 2006; Hassan et al., 2010). There are two hypotheses to explain appearances of *gacS* or *gacA* spontaneous mutants. One hypothesis speculates that these mutants have a reduced metabolic load compared with the wild-type since Gac-mutants often become the major population in the fermentation culture (Jousset et al., 2009). Whereas, Gac-mutants can coexist with wild-type attributing to their high frequency but not predominant in the natural world (Chancey et al., 2002; van den Broek et al., 2005b).

*Pseudomonas chlororaphis* HT66 isolated from the rice rhizosphere is a non-pathogenic strain with wider antagonistic activities against a variety of plant pathogenic fungi due to its main secondary metabolite, i.e., phenazine-1-carboxamide (PCN) (Jin et al., 2016). PCN exhibits noteworthy inhibitory activity against *Fusarium oxysporum, Rhizoctonia solani, Pythiumultimum Trow* and watermelon *Fusarium wilt* (Tupe et al., 2015). Over the past years, our research group has made tremendous efforts at improving the PCN yield through mutation breeding and metabolic engineering for industrial applications. Herein, a spontaneous phenotypic variant (HT66-FLUO) that cannot revert to wild-type appears with high frequency on KB agar plate or in shake flask culture is reported. The strain has obvious phenotypic characteristics such as no PCN production and enhanced fluorescence under UV light. Also, its whole genome sequencing was performed to identify whether there is Single-Nucleotide Polymorphism or InDel in the genome of HT66-FLUO. The differential levels of gene expression of *P. chlororaphis* HT66 and HT66-FLUO was examined using RNA-sequencing, and several pathways and gene regulatory systems were identified to find out the reason related to the phenotypic change of HT66-FLUO.

## Materials and Methods

### Bacterial Strains and Growth Conditions

All the strains, plasmids and oligonucleotide primers used in this study are listed in Supplementary Table S1. The *P. chlororaphis* HT66-FLUO described in our study was isolated from King’s B broth (KB) (Tryptone 20 g, Glycerol 15 ml, MgSO_4_ 0.732 g, K_2_HPO_4_⋅3H_2_O 0.514 g/L) agar plates of *P. chlororaphis* HT66. Briefly, HT66 from -80°C freezer was diluted (10^-7^ ∼ 10^-8^ CFU/ml) and plated on KB agar plates followed by incubation at 28°C for 48 h in a temperature-controlled incubator. After the designated time, the bacteria with larger colonies and no green pigments (PCN) on the surface, were chosen and checked under UV lamp. For sub-culturing, the selected bacteria were diluted and plated on KB agar plates to ensure that colonies are individual. Single colonies of WT or variant were cultured in 5 ml KB liquid medium at 28°C and 180 rpm for 12 h. The cultures were diluted on agar plates and incubated at 28°C for 60 h and HT66-FLUO was isolated. Luria–Bertani (LB) medium (Tryptone 10.0 g, Yeast extract 5.0 g, NaCl 10.0 g/L) was used to cultivate *Escherichia coli* at 37°C. When necessary, antibiotics and other compounds were used at the following concentrations: kanamycin (Km) 50 μg ml^-1^, ampicillin (Amp) 100 μg ml^-1^, isopropyl β-D-1-thiogalactopyranoside (IPTG), 0.1 mM in promoter induction, and *ortho*-nitrophenyl-β-galactoside (ONPG), 4 mg ml^-1^ in β-galactosidase quantification.

### PCR and Sequence Analyses

Standard genetic engineering methods were used according to the standard procedures (Sambrook and Russell, 2001). KOD New Plus (Toyobo) was used for DNA manipulations of plasmid construction and sequence analyses, whereas The Easy Taq DNA polymerase (Transgen) was applied to screen mutant strains. Nucleotides and amino acid alignments were searched on NCBI^1^ and *Pseudomonas* Genome DB^2^.

### Transmission Electron Microscopy (TEM)

Transmission electron microscopy was performed at the Instrumental Analysis Center of Shanghai Jiao Tong University, Shanghai, China. The HT66 and HT66-FLUO bacterial strains were incubated in KB medium for 23 h or 16.5 h, respectively. For TEM analysis, samples were treated with the Sörensen-sucrose phosphate buffer (0.1 M phosphate at pH 7.5, 0.65 M sucrose, 2.5 mM CaCl_2_) containing both 2.5% glutaraldehyde and 1% formaldehyde. The treated samples were placed into TEM grid which was stained with Methylene Blue-Azur II for 5 min and observed with 120kV Biology Transmission Electron Microscope (Tecnai G2 SpiritBiotwin, FEI, United States).

### Determination of Cell Growth and PCN Production

Bacterial growth was monitored by determining the optical density of the culture broth at 600 nm using a double beam UV-vis spectrophotometer (UV-7504, Xinmao, Shanghai, China). For this, 200 μL of fermentation broth was taken into a 1.5 mL Eppendorf tube and centrifuged at 13,000 *g* for 5 min. The resulting residue was dissolved in 1 mL ddH_2_O and diluted appropriately with ddH_2_O to make OD_600_ value between 0.2 and 0.8. The real OD_600_ value was calculated by multiplying the diluted OD_600_ with dilution factor. The growth curve was drawn using the real OD_600_ value.

In order to quantify the PCN, a 400 μL fermentation broth was first acidified to pH 2.0 with 20 μl 6 M HCl, and then 3.6 mL ethyl acetate was added. The samples were vigorously agitated and centrifuged at 13,000 *g* for 5 min. A 400-μL portion of the upper layer was collected and evaporated in a rotary evaporator. The residues containing PCN were dissolved in 1 mL acetonitrile and determined by HPLC (Agilent Technologies 1200 series, Santa Clara, CA, United States) with a C18 reversed-phase column (Agilent Eclipse, XDB-C18, 4.6 mm × 250 mm, 5 μm, Santa Clara, CA, United States) at 254 nm. The mobile phase consists of 92% 5 mM ammonium acetate and 8% acetonitrile and used at a flow rate of 1 mL/min.

### *In Vitro* Assay for Siderophore Production

The quantitative method of siderophore production in *P. chlororaphis* HT66 and HT66-FLUO based on universal chrome azurol S (CAS) was followed by Schwyn and Neilands (1987). An appropriate cell concentration of HT66 or HT66-FLUO was carefully dropped on the middle area of CAS blue agar and the plates were incubated at 28°C for 60 h. The siderophore production was reflected by the diameter of a distinct fluorescent orange zone on the CAS plate.

### Biofilm Formation Assay

Overnight cultures of *P. chlororaphis* HT66 and HT66-FLUO were diluted with 0.01 M phosphate-buffered saline (PBS) to 10^6^ CFU/mL, and 100 μL diluted cultures were seeded on the wells of a 24-well plate with round-bottom. The plate was then incubated at 28°C for 48 h without shaking. After a designated time, the culture was gently removed by pipetting and each well was washed twice with 150 μl 0.01 M sterile PBS. Afterwards, 200 μL of 1% (w/v) crystal violet (CV) was added to each well to stain bacterial biofilm, and the plate was incubated at 28°C for 20 min. The CV was then rinsed with 500 μL 95% ethyl alcohol for 10 min and the amount of biofilm was quantified by measuring the OD_540_ of CV concentration.

### Swarming and Twitching Motility Assays

The swarming and twitching motility assays were performed as described earlier (Rashid and Kornberg, 2000). Swarming motility was determined by inoculating overnight cultures of WT and HT66-FLUO on swarming plates (10 g/L Tryptone, 5 g/L Yeast extract, 5 g/L Glucose, 5 g/L Agar) followed by incubation at 28°C for 20 h. In the twitching motility assays, bacteria were stabbed into twitch agar plates (10 g/L Tryptone, 5 g/L Yeast extract, 5 g/L NaCl, 10 g/L Agar), and the zone of twitching was observed after incubation of twitch plates at 28°C for 30 h.

### RNA-Sequencing

For RNA-sequencing, three HT66 and three HT66-FLUO samples were collected at the late exponential phase (HT66 21 h; HT66-FLUO 16.5 h) by centrifuging at 6000 rpm for 10 min at 4°C. The cell pellets were rapidly washed with pre-chilled PBS and then recollected by centrifugation (3,000 rpm at 4°C for 3 min). The precipitated cells were immediately resuspended in 1 mL of TRIzol reagent (Invitrogen) at room temperature for 20 min and then 200 μL chloroform was added. The samples were vortex mixed for 15 s and centrifuged at 4°C for 15 min. Subsequently, the liquid layer was transferred into a new tube followed by the addition of 480 μL isopropanol. Similarly, the mixed samples were centrifuged at 4,600 × *g* and 4°C for 15 min. The RNA pellets were washed with 70% ethanol, dissolved in RNase-free water, and purified using a Qiagen RNeasy Mini kit. The concentration and purity of RNA were determined by a BioAnalyzer apparatus (Agilent Technologies). After DNaseI treatment, rRNAs were removed from total RNA using the Ribo-Zeror RNA Removal Kit (Bacteria, EPICENTRE). The samples were mixed with fragmentation buffer (Ambion) and then incubated at 70°C. The RNA was fragmented into 130–170 nt and purified with RNAClean XP Beads. The first-strand cDNA was amplified using First-Strand Master Mix and SuperScript II reverse transcription (Invitrogen). The resulting cDNA was used for synthesizing second-strand cDNA with Second-Strand Master Mix. The purified second cDNA strands were added A-tailing for further constructing the sequencing library. The final library was quantitated in two ways: determining the average molecule length using the Agilent 2100 Bioanalyzer instrument, and quantifying the library by qPCR (TaqMan Probe). The qualified libraries were amplified and sequenced on HiSeq 2000 System (TruSeq SBS KIT-HS V3, Illumina). The gene expression abundances of HT66 and HT66-FLUO were calculated by the method of Fragments per kilo-base of mRNA per million reads (FPKM). A False Discovery Rate (FDR) corrected *p*-value ≤ 0.05 and a threshold fold change ≥ 2 were used to denote differentially expressed genes.

### Quantitative Real-Time PCR

Bacterial samples were prepared similarly to RNA-seq preparation. A total of 10 genes (*acsA, copZ, impA, piluA, pvdA, ntiB, moaB, phzR, fusA*, and *shiA*) were selected for qRT-PCR analysis. The total RNAs was extracted from the cells of HT66 and HT66-FLUO using a total RNA isolation reagent (Invitrogen, Carlsbad, CA, United States), and reverse transcribed to cDNA using a TaKaRa RNA PCR Kit Ver.3.0. The resulting cDNA was amplified and quantified by RT-PCR with a Real Master Mix (SYBR Green) RT-PCR Kit (TaKaRa) on ABI Step-One Plus Real-Time PCR system. The *rpoD* gene was used as a reference. The expression level of mRNAs between HT66 and HT66-FLUO was compared by the 2^-ΔΔ^*^C^*^t^ method (Livak and Schmittgen, 2001).

### Genome Sequencing and Identification of Genetic Variations

The DNeasy MiniPrep Kit (Qiagen, China) was used to isolate genomic DNA from bacterial cultures according to the manufacturer’s instructions. The fragmented DNA was incubated at 20°C for 30 min, to combine with End Repair Mix. The purified end-repaired DNA was added A-Tailing using A-Tailing Mix and then incubating the purified Adenylate 3′Ends DNA with Adapter and Ligation Mix at 20°C for 15 min. Adapter-ligated DNA was purified and amplified with Primer Cocktail and PCR Master Mix to construct the library. The final library was quantitated by determining the average molecule length (Agilent DNA 1000 Reagents), and real-time quantitative PCR (qPCR) (TaqMan Probe). The final libraries were then amplified and sequenced on HiSeq 2500 System (HiSeq SBS Kit V4, Illumina). In order to make the subsequent analysis results more accurate and reliable, low-quality data in the Raw Data were removed. The processed data called Clean Data was assembled by SOAPdenovo V2.04 short sequence assembly software. Reads were compared with assembly Contig. According to the reads paired-end and overlap, the results were further optimized and assembled. Using SOAPaligner (version 2.21) sequence alignment software, all reads were mapped to the reference genome of *P. chlororaphis* HT66 (GenBank under the accession number ATBG00000000). The reads were trimmed with a quality threshold of 5 to remove sequences of low read quality or sequences with adapters. According to the comparison results, the sequence coverage was 100% at a depth of 282. The filtered reads are assembled by soapdenovo V1.05, and the resulted scaffolds were mapped to reference genome for further SNP and InDel analysis. Each sample was globally compared with a reference sequence to find out the differences between the query sequence and the reference genome, using MUMmer V 3.22 comparison software, to detect potential SNP sites. To verify the SNP site, the sequence of 100 bp on both sides of the SNP locus of the reference sequence was extracted and compared with the assembly results by BLAST (version 34). The repetitive SNP was removed by filtration with BLAST V2.2.2, TRF V4.04 and Repeatmask V3.2.9 software, and got a reliable SNP. The query and reference sequence were compared using LASTZ V1.01.50 software and results are further optimized with axt_correction, axt_Sort and axt_Best programs to select the best comparison results. Similar to SNPs, the indel results were further verified through BWA V0.5.8 and samtools V0.1.7 software.

### Deletion of *gacA, gacS*, and *pvdA* in *P. chlororaphis* HT66

To construct a non-scar mutant HT66Δ*gacA*, the upstream fragments were amplified with primers *gacA*-F1 (*Xba*I) and *gacA*-R1 and the downstream fragments with primers *gacA*-F2 and *gacA*-R2 (*Hind*III) (see Supplementary Table S1). The 565 bp upstream and 633 bp downstream fragments were further ligated through an overlap PCR based on a 20 bp homology region between the two primers of *gacA*-R1 and *gacA*-F2. Then, the 1198 bp fusing DNA fragment was digested with restriction enzymes *Xba*I and *Hind*III, and cloned into the *Xba*I-*Hind*III-digested pK18mobsacB to generate the recombinant plasmid pK18-*gacA*. The resulting plasmid was transformed into *P. chlororaphis* HT66 from *E. coli* S17-1 (λpir) constructing HT66Δ*gacA* mutant by biparental mating. The single crossover clones were selected on LB plates containing 100 μg/ml Amp and 50 μg/ml Km, whereas the double crossover clones were selected on LB plates containing 100 μg/ml Amp and 15% sucrose. The HT66Δ*gacA* mutant was confirmed by PCR analyses and sequencing. The *gacA* (642 bp) gene was removed from +116 bp to +637 bp (relative to ATG). Using the same method, we knocked out *gacS* (2754 bp) from +75 bp to +2699 bp and *pvdA* (1335 bp) from -7 bp to +1295 bp, respectively (relative to ATG).

### Complementation or Overexpression of *gacA, gacS, rsmX, rsmY*, and *rsmZ*

To complement or overexpress the *gacA, gacS, rsmX, rsmY*, and *rsmZ* genes in HT66 and HT66-FLUO, the expression plasmids pBBR2*gacA*, pBBR2*gacS*, pBBR2*rsmX*, pBBR2*rsmY*, and pBBR2*rsmZ* were constructed as follows. The *gacA* gene was PCR-amplified, digested with restriction enzymes *Xho*I and *Hind*III, and cloned into pBBR1MCS2. Similarly, we amplified *gacS* gene, *rsmX* gene with its 109 bp upstream and 24 bp downstream, *rsmY* gene with its 89 bp upstream and 30 bp downstream and *rsmZ* gene with its 301 bp upstream and 70 bp downstream. The resulting fragments were inserted into pBBR1MCS2. All the genes in pBBR1MCS2 plasmid were transcribed using the *lacZ* promoter.

### Construction of the *lacZ* Fusion Plasmid

To investigate the expression difference of *rsmX, rsmY*, and *rsmZ* between HT66 and HT66-FLUO, the promoter regions of these genes and *lacZ* were cloned into a pBBR1MCS2 plasmid in which the T7 promoter had been terminated by the rrnBT1 terminator. The pBB-*rsmX*-*lacZ* plasmid harboring 109 bp fragment upstream of *rsmX* was constructed into the *XbaI/HindIII* digested plasmid pBBR1MCS2. Similarly, pBB-*rsmY*-*lacZ* and pBB-*rsmZ*-*lacZ* were constructed using the same method, including an 89 bp fragment upstream of *rsmY* and a 301 bp fragment upstream of *rsmZ*.

### Assessment of Urea Utilization

To assess the different ability of urea utilization between *P. chlororaphis* HT66 and HT66-FLUO, the strains were grown in minimal medium with or without the addition of urea. A 20 mM urea as the sole nitrogen source was added into the M9 medium that contains per liter: 5 g glucose, 2.99 g KH_2_PO_4_, 0.58 g NaCl, 0.246 g MgSO_4_⋅7H_2_O, 17.17 g Na_2_HPO_4_⋅12H_2_O, 0.01 mg vitamin B1, 0.6 mg CoCl_2_⋅6H_2_O, 1 mg MnCl_2_⋅4H_2_O, 0.6 mg H_4_MoNa_2_O_6_, 4.3 mg CaCl_2_, 16.7 mg FeCl_3_⋅ 6H_2_O, 1.7 mg ZnCl_2_, 0.43 mg CuCl_2_⋅2H_2_O. *P. chlororaphis* HT66 and HT66-FLUO strains were inoculated into 5 ml LB for 12 h at 28°C. After washing twice with M9 medium, bacteria were transferred into 50 ml of the above-mentioned M9 medium at a final OD_600_ of 0.03 and cultured at 28°C with 180 rpm.

## Results

### Phenotypic Characterization of HT66 and HT66-FLUO

#### Cell Morphology and Growth

**FIGURE 1:**
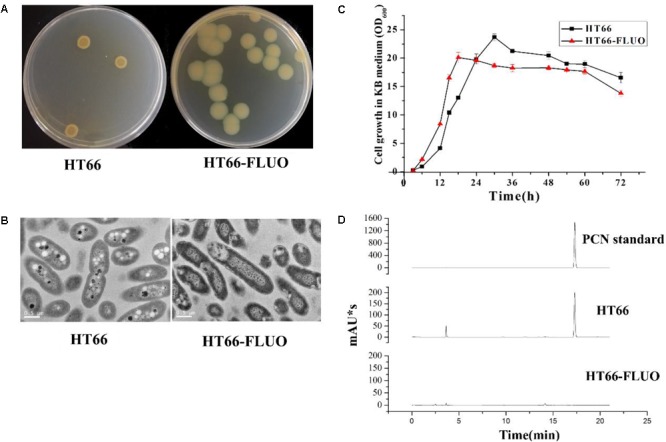
The morphology, growth, and phenazine-1-carboxamide production of *Pseudomonas chlororaphis* HT66 and HT66-FLUO. **(A)** Comparison of single colony of HT66 and HT66-FLUO following the bacterial strains incubation on KB agar plates for 72 h. **(B)** Analysis of cell morphology of *P. chlororaphis* HT66 and HT66-FLUO by Transmission electron microscopy. **(C)** Growth curves of HT66 and HT66-FLUO grown in KB culture medium at 28°C and 180 rpm. **(D)** PCN production of *P. chlororaphis* HT66 and HT66-FLUO in HPLC analysis. The top is 72.5 mg/L PCN standard, Middle is the PCN production of *P. chlororaphis* HT66 and Bottom is the PCN production of mutant strain HT66-FLUOin KB broth at 28°C with 180 rpm for 60 h.

**Figure 1A** shows the colony morphology of *P. chlororaphis* HT66 and HT66-FLUO on KB agar plates after 3 days of incubation at 28°C. Individual colonies of WT strains were observed to be round (∼7.76 ± 0.8 mm in diameter) and smooth with regular margins, while colonies of HT66-FLUO appeared more transparent and larger (∼11.46 ± 1.2 mm in diameter) after subculturing and this phenotype persisted after several cultivation trials. A comparative growth profile of the strain HT66 and HT66-FLUO by monitoring OD_600_ is portrayed in **Figure 1C**. The growth rate of HT66-FLUO increased more rapidly than that of HT66 during log phase, but the cell density was reduced in stationary phase. Morphological differences between HT66 and HT66-FLUO showed that the HT66-FLUO cells were more slender than HT66 (**Figure 1B**).

#### PCN and Siderophore Production Variance in HT66 and HT66-FLUO

**FIGURE 2:**
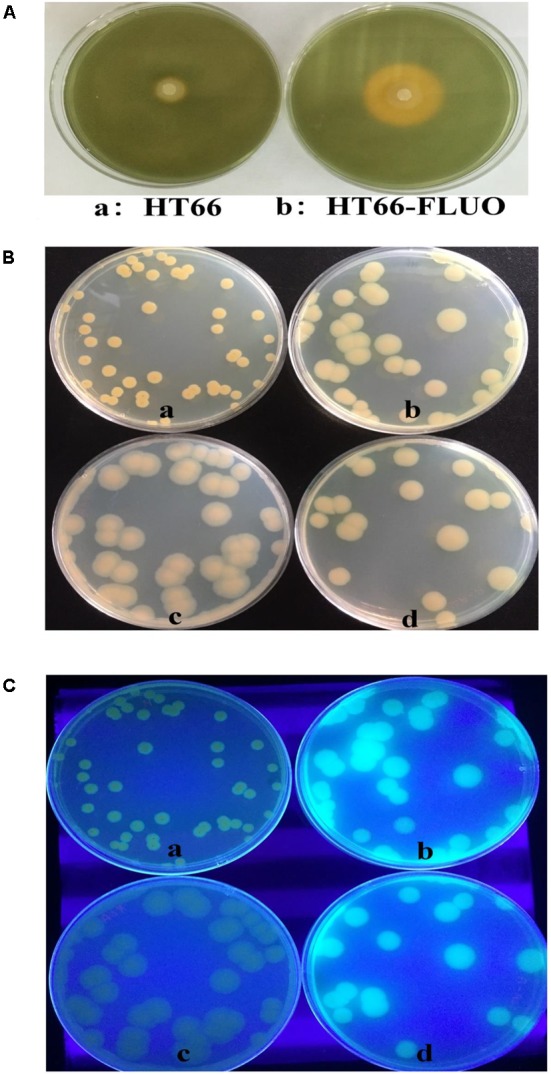
Siderophore biosynthesis assays. **(A)** Determination of siderophore production of *P. chlororaphis* HT66 and HT66-FLUO on CAS blue agar plates. The bacteria were incubated for 60 h at 28°C. The siderophore production is indicated by the size of the orange zone on the center of CAS plates. **(B)** Comparison of the morphology. **(C)** Comparison of the fluorescence phenomenon under UV light. The strains were grown on KB agar plates at 28°C for 60 h. HT66 (a), HT66-FLUO (b), HT66-FLUOΔ*pvdA* (c), HT66-FLUOΔ*pvdA*-*pvdA* (d).

Approximately, 0.42 g/L PCN was produced by HT66 strain after 60 h of fermentation. However, PCN production was disappeared in the culture filtrate of HT66-FLUO (**Figure 1D**). One of the most remarkable features of HT66-FLUO observed was fluorescing under UV light, which makes HT66-FLUO like fluorescent *Pseudomonas* species. There are two gene clusters in *P. chlororaphis* HT66 involved in siderophores synthesis, i.e., pyoverdine and achromobactin (Chen Y. et al., 2015). By sequence analysis, we found the biosynthetic locus of pyoverdine in the HT66 genome, which implied the production of fluorescent pyoverdine in HT66-FLUO. Therefore, we first measured the siderophores production of strain HT66 and HT66-FLUO on CAS solid plates. The diameter of the yellow zone of the chelated halo was significantly wider in HT66-FLUO than WT (**Figure 2A**). The siderophores production was substantially increased in HT66-FLUO. To confirm the cause of fluorescence in HT66-FLUO, we constructed a deletion mutant of *pvdA*, which is an indispensable part of pyoverdine biosynthetic gene cluster. As shown in **Figure 2B**, the yellow–green fluorescence disappeared completely in *pvdA-* mutant. However, on insertion of *pvdA* gene in HT66-FLUO (*pvdA-*) chromosome, the fluorescence ability of strain HT66-FLUO restored (**Figure 2C**). These results indicate that fluorescent substance was pyoverdine, and the phenomenon of fluorescence in HT66-FLUO relates to the increased production of pyoverdine.

### Genome Variations in HT66 and HT66-FLUO

Since the phenotype of HT66-FLUO was stable, we identified whether there exist Single-Nucleotide Polymorphisms (SNPs) or InDel in the genome of HT66-FLUO. Compared to the reference genome of HT66, 34 SNPs were identified including 20 synonymous and 14 non-synonymous mutations. In addition, 8 insertional mutations, 3 deletion mutation were detected in HT66-FLUO genome (Supplementary Tables S7, S8). Interestingly, when we verified the correctness and repeatability of the whole genome resequencing data, we cannot obtain the repeatability results from PCR product amplifying from HT66 and HT66-FLUO genome.

### Global Gene Expression Profiles in HT66-FLUO

**FIGURE 3:**
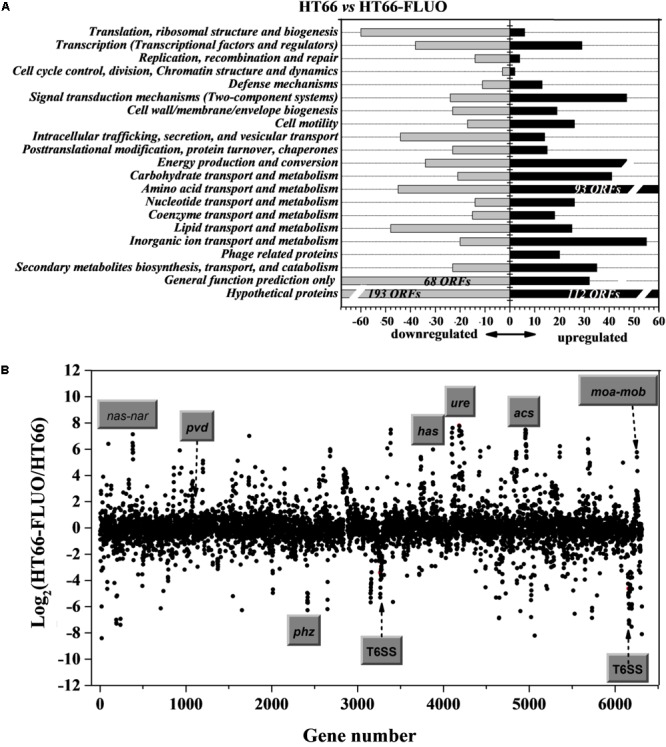
**(A)** Functional classification of differential levels of gene expression with a fold change of at least 2.0 was performed according to *P. chlororaphis* HT66 in NCBI database. Gray and black bars represent the number of significantly downregulated and upregulated genes, respectively. **(B)** Scatter plot comparing the transcriptomes of *P. chlororaphis* HT66 and HT66-FLUO. The *x*-axis shows gene number in the HT66 genome; the *y*-axis shows the log_2_ of fold change of transcript abundance of each gene in the HT66-FLUO relative to HT66. The highly modulated and function-known gene clusters are marked.

The transcriptome sequencing experiment was utilized to determine the causes for the phenotypic modulation observed in *P. chlororaphis* HT66 and HT66-FLUO, because the RNA-sequencing is a direct measurement of different gene expression levels, and is more sensitive than DNA microarray. For this, we compared the transcriptional profile of HT66 and HT66-FLUO grown in KB at 21 and 16 h in logarithmic phase, respectively. Compared with HT66 strain, 1,418 genes in HT66-FLUO, which represented approximately 22% of the 6393 open reading frames (ORFs), showed the significant difference (increased or decreased by at least twofold; *P* < 0.05) on transcriptional levels. Among them, 679 genes were upregulated and 739 genes were downregulated (**Figure 3A**). The results showed a comprehensive transcription rearrangement in HT66-FLUO strain. Scatter plot showed the transcriptomes of *P. chlororaphis* HT66 and HT66-FLUO (**Figure 3B**).

**FIGURE 4:**
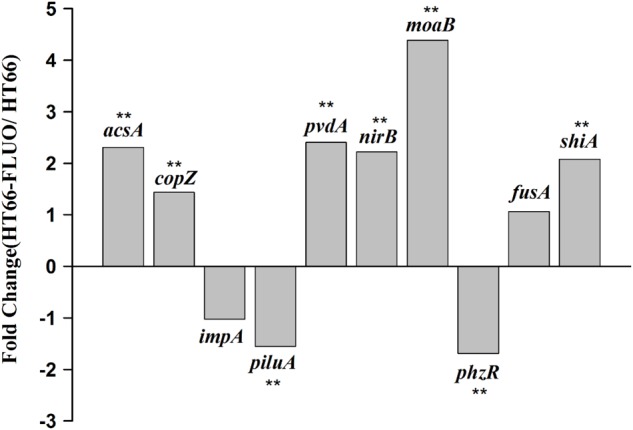
Validation of Transcriptional data using RT-qPCR assays. Student’s *t*-test was carried out for the statistical analyses of the data and “^∗∗^” represents statistically significant difference at *p <* 0.01.

The data validation of gene expression difference obtained by transcriptome sequencing was performed through qRT-PCR. The selected 10 genes: *acsA*, which encodes achromobactin biosynthesis protein; *copZ*, which is an unknown-function gene but conserved in bacteria; *impA*, encoding components of the type VI secretion systems; *piluA*, which encodes membrane protein and is related to bacteria motility; *pvdS*, participating in pyoverdine biosynthesis; *nirB*, which encodes protein that related to nitrogen utilization; *moaB*, encoding proteins that involved in coenzyme transport and metabolism; *phzR*, which encoding components of the quorum-sensing system and can regulate PCN biosynthesis; *fusA*, which encodes an elongation factor related to SCV phenotype; *shiA*, which encodes a regulatory factor involved in DHS transport. The qRT-PCR results were comparable to those from RNA-seq (**Figure 4**).

### Changes in PCN Biosynthesis

**FIGURE 5:**
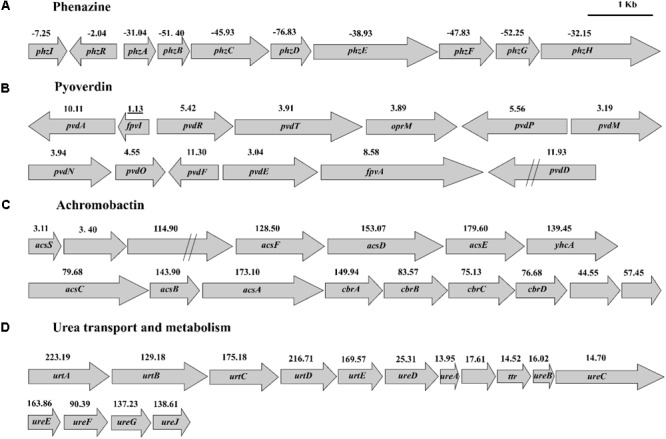
Transcription levels of gene clusters of PCN synthesis **(A)**, pyoverdin synthesis **(B)**, achromobactin synthesis **(C)**, and urea transport and metabolism **(D)**.

The expression of *phzI/phzR* genes which are part of quorum sensing system and directly regulate PCN biosynthesis were found to be downregulated by 7.25- and 2.04-fold, respectively (**Figure 5A**). The transcript levels of genes in PCN biosynthetic operon were significantly downregulated (from 31.04- to 76.83-fold) (**Figure 5A**). The gene expression of the *rpeA-rpeB* system was downregulated, while the *gacs-gacA* system remained unchanged. RpeA is a negative regulation factor and RpeB is a positive regulator of PCN synthesis in HT66, and PCN production decreased in *rpeA-rpeB* double mutant (Unpublished data). The gene expression of other regulatory factors was upregulated or downregulated in HT66-FLUO compared to wild-type HT66 (see Supplementary Table S2).

### Changes in Iron Uptake

Genes involved in iron homeostasis were significantly upregulated in HT66-FLUO. According to genomic analysis, HT66 produces two siderophores, pyoverdine, and achromobactin. Pyoverdine synthesized from amino acid precursors by non-ribosomal peptide synthetase (NRPS) provides the fluorescent *Pseudomonas* species with their defining fluorescent and yellow–green pigmentation under iron-limited conditions (Visca et al., 2007). Achromobactin is synthesized by a mechanism entirely independent of NRPS enzymes. As shown in **Figure 5B**, the transcription level of genes associated with pyoverdine biosynthesis, regulation and transport were significantly higher in HT66-FLUO than HT66 that is consistent with hyper-fluorescence of HT66-FLUO. The genes of achromobactin biosynthesis cluster were overexpressed by 44.5–79.6-fold in HT66-FLUO (**Figure 5C**). In addition to siderophore, several pathogens utilize heme as an iron source, including the opportunistic *P. aeruginosa*, which encodes two heme uptake systems; *Pseudonmonas* heme uptake (Phu) and heme assimilation (has) systems (Kaur et al., 2009; Smith et al., 2015). The HT66 homolog of heme acquisition system A (HasA), known as a hemophore in several Gram-negative pathogens including *P. aeruginosa*, was upregulated by 63.1-fold in HT66-FLUO. Moreover, the related transport and regulator genes were upregulated by 3.1–17.9-fold. Alternatively, the genes in heme-uptake pathway increased 1.5–4.0-fold, which encodes a TonB-dependent outer-membrane (OM) receptor (PhuR) that transports heme to the periplasm (Smith et al., 2015), where a soluble heme-binding protein (PhuT) acts as the receptor for an ATP-dependent permease (ABC transporter) (Phu UV) (see Supplementary Tables S3).

### Changes in Secretion Systems

Bacteria use multiple protein secretion systems (SS) for pathogenesis, niche adaptation, and utilization of nutrients (Ma et al., 2003). Transcriptomic data determined that the multiple genes in secretion system were differentially expressed. Type II secretion system (T2SS) related genes were upregulated (Supplementary Table S4), especially, VreR as an anti-σ factor in *vreAIR* operon (Quesada et al., 2016), showed significant upregulation (7.77-fold) in comparison with other genes in T2SS gene cluster. However, another newly defined secretion system T6SS that is structurally and mechanistically analogous to an intracellular membrane-attached contractile phage tail, was markedly downregulated at the level of transcription in HT66-FLUO. In the present study, RNA-sequencing indicated that three distinct homologs H1-T6SS, H2-T6SS, and H3-T6SS were differentially expressed. In *P. aeruginosa*, the expression of H1-T6SS gene is regulated by the RetS, which is -2.27-fold in HT66-FLUO. Therefore, the genes in H1-T6SS operon were 10.1–158.2-fold downregulated. Three gene clusters of T6SS were significantly downregulated at the transcript level in HT66-FLUO in which PCN is nearly unproduced compared with HT66.

### Differential Expression of Genes Involved in Swarming Motility, Twitching Motility, and Biofilm Formation

**FIGURE 6:**
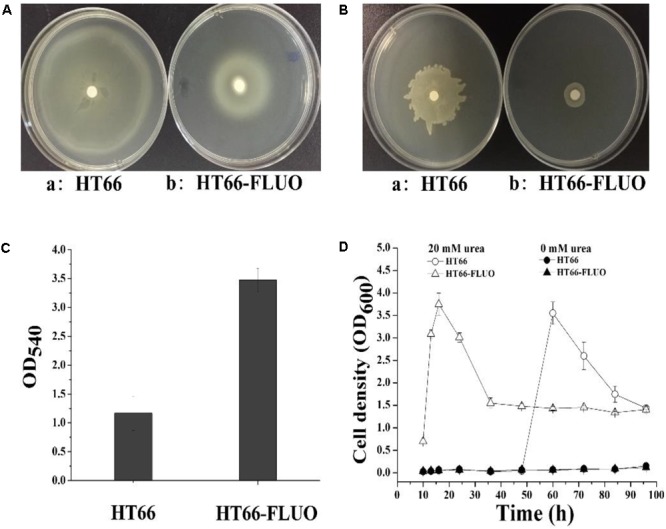
**(A)** Twitching motility (1% agar) and **(B)** swarming motility (0.5% agar) of *P. chlororaphis* HT66 and HT66-FLUO that incubated at 28°C for 20 h. **(C)** Biofilm formation by *P. chlororaphis* HT66 and HT66-FLUO. **(D)** Growth of HT66 and HT66-FLUO in liquid M9 medium with or without urea as sole nitrogen source.

*Pseudomonas* motility is conducive to adjusting to a different environment, surface attachment, and biofilm formation. Motility allows bacteria to colonize different environments, attach to surfaces, and form biofilms. In our RNA-seq data, multiple genes related to motility were differentially affected. The swarming motility and twitching motility of HT66 and HT66-FLUO were measured on a plate to determine the difference in motile ability between two strains. As shown in **Figures 6A,B,** the swarming and twitching motility of HT66-FLUO strain were weaker than that of HT66 since HT66-FLUO forms smaller and less regular bacterial motility circles on twitching motility plate than HT66. In *Pseudomonas*, motility is related to flagella biosynthesis, pili, and chemotaxis (Rashid and Kornberg, 2000). Interestingly, four genes in the flagella biosynthesis cluster were upregulated by almost 2-fold, and 9 genes annotated as pile biosynthesis cluster were 3–15-fold upregulated (Supplementary Table S6). In *P. chlororaphis* 30–84, the upregulated type IV pili biosynthesis cluster was related to greater adherence ability (Wang et al., 2015). However, 17 genes related to motility were downregulated by 2–31-fold (Supplementary Table S6). Previous work mentioned that the attachment ability of the mutants, such as SCVs, altered compared with WT strain, therefore the biofilm forming ability of HT66-FLUO and HT66 was determined using a CV staining method. The results showed that the biofilms formed by HT66-FLUO were threefold higher than HT66 (**Figure 6C**).

#### Change in Urea Transport and Metabolism

The transcriptions of urea transport and metabolism clusters were significantly (3-223 times) enhanced in HT66-FLUO (**Figure 5D** and Supplementary Table S5). The results in **Figure 6D** showed that HT66 and HT66-FLUO hardly grew in the M9 media without urea. When 20 mM urea was added into M9 media as the nitrogen source, HT66-FLUO grew rapidly in the first 16 h, with OD_600_ up to 3.8. The cell growth of HT66 was significantly inhibited before 48 h of culture and reached a maximum OD of 3.5 at 60 h. The result demonstrated that the capability of HT66-FLUO to utilize urea is obviously superior to HT66.

### Gac-Rsm Systems Regulate the Production of PCN and Fluorescent Siderophore in HT66-FLUO

In order to find the variations in HT66-FLUO genome, the genome of HT66-FLUO was re-sequenced, but we did not get any effective point mutation. The GacS/GacA two-component system could regulate the production of secondary metabolites, such as positive regulation of phenazine production in *P. chlororaphis* 30–84, non-fluorescent siderophore biosynthesis in *Pseudomonas* sp. strain HYS, negative regulation of phenazine production in *P. aeruginosa* M18. Therefore, we knocked out the *gacA* gene in the HT66 strain, constructing HT66Δ*gacA*. The colony morphology of HT66Δ*gacA* was similar to that of HT66-FLUO, but its colony color was white, while HT66-FLUO colony was slightly green. Under UV conditions, both HT66Δ*gacA* and HT66-FLUO had strong fluorescence. The colony morphology of HT66Δ*gacA* was recovered to wild-type by complementary exogenous *gacA* gene. On overexpressing *gacA* gene in the HT66-FLUO, its colony morphology cannot be restored to HT66.

**FIGURE 7:**
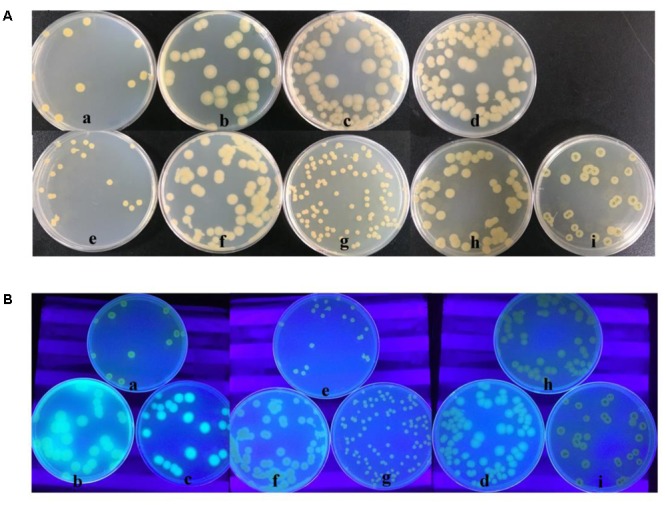
The Comparison of morphology **(A)** and the fluorescence phenomenon under UV light **(B)** among HT66 (a), HT66-FLUO (b), HT66Δ*gacA* (c), HT66Δ*gacS* (d), HT66-pBBR1MCS2 (e), the overexpressing *gacA* strain, HT66-FLUO-pBBR-*gacA* (f), the complemented *gacA* mutant strain, HT66Δ*gacA*-pBBR-*gacA* (g), the complemented *gacS* mutant strain, HT66Δ*gacS*-pBBR-*gacS* (h), the overexpressing *gacS* strain, HT66-FLUO-pBBR-*gacS* (i).

**FIGURE 8:**
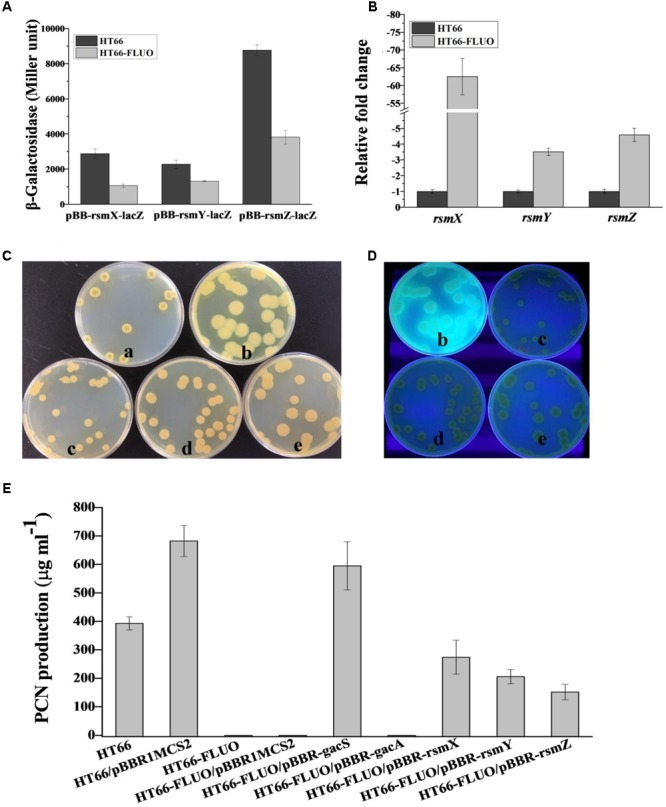
**(A)** β-Galactosidase measurements were performed on expression from the pBB-*rsmX*-*lacZ*, pBB-r*smY*-*lacZ*, pBBR-*rsmZ*-*lacZ* transcriptional fusion plasmid in the wild-type strain HT66 and HT66-FLUO in KB medium. **(B)** The transcript levels of *rsmX, rsmY, rsmZ* in *P. chlororaphis* HT66 and HT66-FLUO determined by qRT-PCR at logarithmic phase. **(C)** The morphology and **(D)** fluorescence phenomenon under UV light of HT66 (a), HT66-FLUO (b), HT66-FLUO/pBBR-*rsmX* (c), HT66-FLUO/pBBR-*rsmY* (d), HT66-FLUO/pBBR-*rsmZ* (e), all the strains were incubated on KB agar plates for 60 h **(E)**, PCN production was determined using HPLC analysis in KB broth at 28°C with 180 rpm for 60 h.

As a two-component system, the GacS kinase can monitor an unidentified environmental signal or condition, and then activates GacA through phosphorylation. Only phosphorylated GacA becomes active, such as activating transcription of *rsmX/Y/Z*. Although the colony morphology of HT66Δ*gacA* was similar to HT66-FLUO, overexpressing *gacA* gene in the HT66-FLUO could not be restored to wild-type levels. According to the transcriptome data, the expression level of *gacA* in HT66-FLUO changed little compared with HT66 (Supplementary Table S1). GacA phosphorylation or other post-translational modification was speculated to be blocked in HTT66-FLUO. So, we overexpressed *gacS* gene in the HT66-FLUO and constructed *gacS* mutant, HT66Δ*gacS*. The colony morphology of HT66Δ*gacS* was similar to HT66Δ*gacA*, the color of HT66Δ*gacS* observed to be transparent than that of HT66-FLUO, and the colony morphology was similar to that of HT66 by complementing exogenous *gacA* gene. As predicted, overexpressing *gacS* in HT66-FLUO restored the biosynthesis of PCN, but overexpression of *gacA* did not restore it (**Figures 7A, 8E**). On the other hand, the strains overexpressing exogenous *gacS* had no fluorescence under UV light (**Figure 7B**).

GacS/A two-component system could activate *rsmX/Y/Z*. However, the expression variation data of sRNAs, *rsmX*/*Y/Z*, were not acquired based on the transcriptome data. Herein, we constructed pBB-*rsmX*-lacZ, pBB-*rsmY*-lacZ, and pBB-*rsmZ*-lacZ plasmids coupled with RT-PCR to detect the relative levels of *rsmX*/*Y/Z*, respectively, both in HT66 and HT66-FLUO. The qRT-PCR result showed that the expression of *rsmX* gene decreased by 65 times, while expressions of *rsmY* and *rsmZ* downregulated by 3–4 times, respectively (**Figure 8B**). The determination result of β-Galactosidase was consistent with qRT-PCR and showed that the level of *rsmX/Y/Z* in HT66-FLUO decreased 2–3 times, relative to the HT66, respectively (**Figure 8A**). Interestingly, the HT66-FLUO colony morphology was restored to wild-type to some extent by overexpressing *rsmX/Y/Z*, separately. The overexpression of *rsmX* transformed the colony morphology of HT66-FLUO to wild-type, the overexpression of *rsmY* led to a transient state between HT66 and HT66-FLUO, and the overexpression of *rsmZ* generated a colony morphology which resembles HT66-FLUO (**Figure 8C**). Similarly, the strains overexpressing exogenous *rsmX, rsmY*, or *rsmZ* had no or slight fluorescence under UV conditions (**Figure 8D**). *RsmX/Y/Z* is able to activate PCN biosynthesis, and expression of *rsmX/Y/Z* in HT66-FLUO obviously decreased. The PCN yields of strains were analyzed by overexpressing *rsmX/Y/Z* in HT66-FLUO; the overexpression of *rsmX, rsmY*, or *rsmZ* in HT66-FLUO resulted in PCN production recovery to some extent, among which the overexpressing *rsmX* in mutant showed the best efficiency (**Figure 8E**). Thus, it was inferred that GacS/A could positively control PCN biosynthesis and negatively control Pvd synthesis by activating the transcription of *rsmX/Y/Z*.

## Discussion

A spontaneous phenotypic variant HT66-FLUO was isolated on KB agar plates or in shake flask cultures of *P. chlororaphis* HT66. The HT66-FLUO was first detected to have non-production of the main secondary metabolite, i.e., PCN but shows strong fluorescence. The strain HT66-FLUO was semi-transparent and without visible PCN on KB agar plates, while the fluorescent siderophore and pyoverdine yield of HT66-FLUO increased significantly. There was a great glistening spherical material in the cytoplasm of HT66 that deduced to be polyhydroxyalkanoates (PHAs) and vanished in HT66-FLUO cells (**Figure 1B**). PHAs are intracellular energy and carbon storage compounds accumulated by several groups of bacteria under unbalanced growth, excessive carbon source or at least the depletion of any other growth essential nutrient (Hartmann et al., 2006; Poblete-Castro et al., 2012).

The *pvdA* knockout-complemented and RNA-seq experiment revealed that the fluorescing phenomenon of HT66-FLUO under UV light was due to the increased pyoverdine yield. *Pseudomonas* secretes different types of siderophores to acquire iron that is crucial for all organisms to maintain a normal life when the available iron is insufficient in the survival environment (Cornelis, 2010). In addition to assisting competition between strains and fitness of *Pseudomonas* strains in a wide variety of environments, earlier studies in our lab showed that enhanced siderophore pathway is related to increased PCN production in wild-type HT66 (Jin et al., 2016). However, pyoverdine and achromobactin production are markedly increased while PCN is abolished in the strain HT66-FLUO indicating that complicated regulatory networks regulate the siderophore pathway.

The phenotype of HT66-FLUO was found to be stable after several passages of cultivation. We speculated that there are Single-Nucleotide Polymorphisms (SNPs) and InDel in the genome of HT66-FLUO since classical evolutionary theory introduces that genetic variation provides the major source of heritable variation. However, we did not find any genetic variation in the HT66-FLUO genome according to the known reference sequence. To date, a variety of molecular mechanisms underlying phase variation are known such as slipped-strand mispairing, genomic rearrangements, spontaneous mutations and epigenetic mechanisms-methylation (van den Broek et al., 2005a). There are also many studies demonstrating that the genetically identical cells present substantial heterogeneity in gene expression, cellular morphology, and physiology (Mondragón et al., 2006; Humair et al., 2009). The phenotypic heterogeneity of different forms including stochastic gene expression variability, alternative protein conformations, morphological plasticity, and cellular age-correlated phenotypic plasticity, can accelerate adaptive evolution. Bacterial populations can respond phenotypically by environment-driven flexible changes in the transcriptional profiles (phenotypic plasticity) and adapt to selective pressures through the process of evolution (evolutionary adaptation) to benefit in distinct habitats (Elena and Lenski, 2003). To further study the causes of the phenotypic variation between HT66 and HT66-FLUO, the transcriptome sequencing experiment was performed.

The production of PCN is controlled by various regulatory factors related to many environmental factors and mineral nutrients (Chin-A-Woeng et al., 2005; Girard et al., 2006). In RNA-sequencing data, all genes expressed in the PCN biosynthetic operon were downregulated. Quorum sensing (QS) is a direct regulation and control way for secondary metabolites in Gram-negative bacteria. In *P. chlororaphis* 1391, PCN biosynthesis is related to quorum sensing system, since it was detected in the high-density period, then phenazine gene cluster was activated after acylated homoserine lactone (AHL) signals bound with PhzR protein (Chin-A-Woeng et al., 2001). In *P. chlororaphis* 30-84, PCL 1391 and HT66, the homologous genes of quorum sensing are *phzI*/*phzR* located in the upstream of the phenazine biosynthetic gene cluster. As gene expression of *phzI* and *phzR* was down-regulated by 7.25- and 2.04-fold, respectively, it directly resulted in down-regulated expression of all phenazine biosynthetic gene clusters.

In *Pseudomonas*, there are multiple two-component signal transduction systems (TCSTS) that influence the production of phenazine and its derivatives (Wang et al., 2013; Li et al., 2015). The expression of genes in GacA/GacS TCSTS and ParR/ParS TCSTS was not different in the HT66-FLUO relative to HT66. However, another TCSTS, RpeA/RpeB can regulate PCN biosynthesis with entire antagonistic effect. *rpeA* and *rpeB* are considered as negative and positive control factors of phenazine synthesis, respectively. In HT66-FLUO, RpeA and RpeB were downregulated in RNA-seq, this is consistent with the result (unpublished) that double mutant of *rpeA*/*rpeB* can result in diminished PCN production in HT66. PCN production is related to the intracellular secretory system that plays an important role in transporting phenazines into the culture medium during fermentation. The type VI secretion system is inferred to control many pathogeneses or non-pathogenesis related phenotypes. Further analyses showed that this system is involved in multiple functions, including antibacterial activity, interactions with other organisms and biofilm formation (Chen L. et al., 2015; Gallique et al., 2017). T6SS is tightly regulated by a wide variety of environmental signals (i.e., temperature, cell density), two-component regulatory system, quorum sensing system, sigma factors (RpoN), histone-like proteins, and post-translational modification (Chen L. et al., 2015). However, the concrete functions and regulatory network of T6SS in *P. chlororaphis* are still not clear. In our RNA-seq data, the gene cluster of type VI secretion system was obviously downregulated. According to our previous study, a high PCN-producing strain, *P. chlororaphis* P3, an integrated strain of mutagenesis and selection, some protein expression of type VI secretion system was enhanced as compared to HT66 (Jin et al., 2016), indicating that type VI secretion system is an important factor for PCN production.

The phenotypic variation of HT66-FLUO was assumed to relate to mutation or deletion of *gacS*/*A*. However, the whole-genome, and PCR-sequencing revealed no mutation of *gacS/A*. The relative expression of *gacS* and *gacA* in WT strain and the variant was almost the same. When comparing the phenotype of strains that are complementing *gacA* in HT66Δ*gcaA* and overexpressing *gacA* in HT66-FLUO on KB agar plates, it was found that the complementing *gacA* in *gacA* knockout strain can revert to wild-type while overexpressing *gacA* in HT66-FLUO cannot. However, the result shows that the cause of phenotypic variation of HT66-FLUO is not *gacA* mutant. Though overexpressing *gacA* did not work in HT66-FLUO, we overexpressed *gacS*, resulting in the recovery of its colony morphology and PCN production to wild-type. However, the *gacS* gene had no any mutation in HT66-FLUO. GacS function in HT66-FLUO was inhibited, such as the formation of two dimers with the RetS protein, and so on, resulting in that GacA could not be phosphorylated (Chambonnier et al., 2016). When *gacS* was overexpressed, excessive GacS kinase could monitor an environmental signal and phosphorylated GacA to activate sRNA transcription. When *rsmX, rsmY*, and *rsmZ* were overexpressed in the strain HT66-FLUO, three sRNAs can partially restore the PCN production as compared with HT66 (**Figure 8E**). It is speculated that *rsmX, rsmY*, and *rsmZ* are involved in regulating the PCN synthesis in *P. chlororaphis* HT66. This is in accordance with an earlier report that Gac system positively controls the expression of non-coding RNA to restrain the RNA-binding protein *RsmE* and activate the signal molecule and PCN synthesis in *P. chlororaphis* 30-84 (González et al., 2008; Lalaouna et al., 2012; Wang et al., 2013). Overexpressing three sRNAs can make the phenotype (colony size, fluorescent under UV light, biofilm and so on) of HT66-FLUO switch to wild-type in different degree; especially reduce the yield of fluorescent siderophore of HT66-FLUO. In *Pseudomonas* sp. strain HYS, after knocking out of *rsmY* and *rsmZ*, the yield of siderophore units was markedly reduced (Yu et al., 2014). Gac and Rsm systems are involved in the regulation of PCN and siderophore biosynthesis in *Pseudomonas*. However, the overexpression of *rsmX* in HT66-FLUO enhances the switching of colony phenotype most significantly as compared to two other sRNAs. It is demonstrated that *rsmX* plays more important effect on phenotype and the expression of secondary metabolites related genes in *P. chlororaphis*. The probable cause of mutant appearance may be related to Gac and Rsm systems. Certain transcription factors are activated to enhance gene expression, and specific signal transduction pathways are induced to adapt to environmental changes, for instance, oxidative, hyperosmotic, thermal, acid, and organic solvent stresses (Guan et al., 2017). In *P. fluorescens* CHA0, the environmental temperature can influence the output of pathways linked with Gac and Rsm systems by influencing the RetS output (Humair et al., 2009). The cause for the development of phenotypic variant HT66-FLUO requires further investigation.

### Nucleotide Sequence Accession Number

The Whole Genome Shotgun Project of HT66 has been deposited in DDBJ/EMBL/GenBank under the accession number ATBG00000000.

## Author Contributions

YL carried out the experimental-based work and drafted the manuscript. HH, WW, XH, HP, and XZ analyzed and interpreted the data. ZW and MB assisted in experiments and drafted the manuscript. All the research work was carried out under the dynamic guidance and supervision of XZ who designed, conceived, and coordinated the experiments. All authors read and approved the final manuscript.

## Conflict of Interest Statement

The authors declare that the research was conducted in the absence of any commercial or financial relationships that could be construed as a potential conflict of interest.

## Footnotes

1 http://www.ncbi.nlm.nih.gov/blast

2 http://www.pseudomonas.com/
