# Supplementary material for: Enhanced Fluorescent Siderophore Biosynthesis and Loss of Phenazine-1-Carboxamide in Phenotypic Variant of Pseudomonas chlororaphis HT66
Source: Front Microbiol. 2018 Apr 23;9:759. doi: 10.3389/fmicb.2018.00759 (PMC5924801; doi:10.3389/fmicb.2018.00759)
Supplement: Supplementary file 1 [file Data_Sheet_1.DOCX]

**Supplementary material**

**Enhanced fluorescent siderophore biosynthesis and loss of phenazine-1-carboxamide in phenotypic variant of *Pseudomonas chlororaphis* HT66**

Yang Liu^1^, Zheng Wang^1^, Muhammad Bilal^1^, Hongbo Hu^1,2^, Wei Wang^1^, Xianqing Huang^1^, Huasong Peng^1^, and Xuehong Zhang^1*^

^1^State Key Laboratory of Microbial Metabolism, School of Life Sciences and Biotechnology, Shanghai Jiao Tong University

^2^National Experimental Teaching Center for Life Sciences and Biotechnology, Shanghai Jiao Tong University, Shanghai, 200240, China

^*^Corresponding author e-mail: [xuehzhang@sjtu.edu.cn](mailto:xuehzhang@sjtu.edu.cn); Tel.: +86-21-3420-6715; Fax: +86-21-3420-5081

**Table S1: Plasmids and primers used in the present study**

| Primer names | Primer sequences (5’-3’) | Applications |
| --- | --- | --- |
| RT-PCR |  |  |
| AcsA-F | CTGACGCTGTTCCTCGAAGT | *acsA* gene |
| AcsA-R | TACTGCTGGTATTCGGCCTG | *acsA* gene |
| CopZ-F | GCGGCCTATTTCGTCATTCAC | *copZ* gene |
| CopZ-R | GCTGCATCTTCATCAGGTCG | *copZ* gene |
| ImpA-F | TTCTCCAGCGAATACGAGGC | *impA* gene |
| ImpA-R | GCGCTTCACTGTTTTCGAGG | *impA* gene |
| PiluA-F | TTGGCACCTTTCTCACTCCG | *piluA* gene |
| PiluA-R | GGATCGCAACCGAATAGGGA | *piluA* gene |
| PvdA-F | TGATCGAACGCATCTACGGG | *pvdA* gene |
| PvdA-R | GTAGTGACGGGTGCTCAACT | *pvdA* gene |
| PstS-F | CACAAGGACAACCCGATCCA | *pstS* gene |
| PstS-R | CGGATACCGAGTTACGACCG | *pstS* gene |
| Nti-F | TCGACGAGGTCAAGAAGCAC | *nti* gene |
| Nti-R | TTTCGCTTTTCGGCTTGACG | *nti* gene |
| Moa-F | GTGCCGGACGACATCTACC | *moa* gene |
| Moa-R | TGCCGATTTCTTCCACCGAC | *moa* gene |
| PhzR-F | CGGTGAAGCACAGCAAAGTC | *phzR* gene |
| PhzR-R | GCAAACGCCTTGGTCATCAG | *phzR* gene |
| FusA-F | CATCTCGGGTATGGGTGAGC | *fusA* gene |
| FusA-R | GGTGATCTTCTCGCGGTAGG | *fusA* gene |
| ShiA-F | GAACATTGCGCTGATGACCC | *shiA* gene |
| ShiA-R | AGGCCAGTTGATAGGCGATG | *shiA* gene |
| RpoD-F | GCTGTGGAAACCGACATTGG | *rpoD* gene |
| RpoD-R | TGACGCGAGTGTATTCGGAG | *rpoD* gene |
| GacA-F | TCAAGTCATTCCAGCCGTCC | *gacA* gene |
| GacA-R | TCTGGCAGCCGACAATCATC | *gacA* gene |
| GacS-F | GGGCGGTTATTTCACCTGGA | *gacS* gene |
| GacS-R | GATACGTTCCAGTAGGGCGG | *gacS* gene |
| rsmX-F | CAACTGCAGCGCAGGAC | *rsmX* gene |
| rsmX-R | CAAGACCATTACGACATCCTGT | *rsmX* gene |
| rsmY-F | GCAGGAAGCGCAAAGCAATA | *rsmY* gene |
| rsmY-R | GGGGCTTTGCAGACTGTTTC | *rsmY* gene |
| rsmZ-F | TCGACGGATAGACACAGCCA | *rsmZ* gene |
| rsmZ-R | TTCCCTAGTCCCTGTGTTCCT | *rsmZ* gene |
| GacA-F1 | CCGGGGATCCTCTAGAATTTGGTGAAGTTGGCGGCGT | *gacA* deletion |
| GacA-R1 | GATTCCTCCCCTGACTCGGCCT | *gacA* deletion |
| GacA-F2 | CGAGTCAGGGGAGGAATCCCTGACAATGACCGACCCGTT | *gacA* deletion |
| GacA-R2 | GGCCAGTGCCAAGCTTAGTTCATCGGTCAGGGCATTG | *gacA* deletion |
| GacA-F1 | CCGGGGATCCTCTAGAGGCTTGGGCAGATAATCGTCG | *gacS* deletion |
| GacA-R1 | ACCAACGCCATCAGGCTGGTT | *gacS* deletion |
| GacA-F2 | CAGCCTGATGGCGTTGGTCGAACTCGAACGGGCAATCAA | *gacS* deletion |
| GacA-R2 | GGCCAGTGCCAAGCTTTTGCGGGTTGGGGAAGGGA | *gacS* deletion |
| PvdA-F1 | CCGGGGATCCTCTAGAAGATGTGGTGCATGACGCTTACG | *pvdA* deletion and complementation |
| PvdA-R1 | GTCCTCTGGATAAATCCCTCAACGC | *pvdA* deletion |
| PvdA-F2 | GGGATTTATCCAGAGGACCCGTTCGGTGCGTGATCTGTT | *pvdA* deletion |
| PvdA-R2 | GGCCAGTGCCAAGCTTCTGTTGCAGTGTCGATGAGAAGGA | *pvdA* deletion and complementation |
| *gacA*-pBbR-F | CCCTCGAGGGAGGAGGCCATCCTTGATTAGGGTGCTAGTAGTCGAT | *gacA* complementation and overexpression |
| *gacA*-pBbR-R | CCAAGCTTGGTCAGGCGCTGGCATCGACC | *gacA* complementation and overexpression |
| *gacS*-pBbR-F | CCCAAGCTTAGGAGGCCATCCGTGCTTAAGAAATTGGGGATCA | *gacS* complementation and overexpression |
| *gacS*-pBbR-R | CGGGATCCTCAGGCGTTGATGCGAGCCT | *gacS* complementation and overexpression |
| *rsmX*-pBbR-F | CGGAATTCCGTGGGTTTCGGGGCCTAGGG | *rsmX* overexpression |
| *rsmX*-pBbR-R | CGGGATCCCGGCCCCAGGGCTTTTCCACA | *rsmX* overexpression |
| *rsmY*-pBbR-F | CGGAATTCCGGTACATGGGCGTCATGTAAGCATT | *rsmY* overexpression |
| *rsmY*-pBbR-R | GCTCTAGAGCAGCAGCGCAGATAAAAAACGCG | *rsmY* overexpression |
| *rsmZ*-pBbR-F | CGGAATTCCGCGGTACGAGCAATCCTGCCGT | *rsmZ* overexpression |
| *rsmZ*-pBbR-R | CGGGATCCCGTCACCTCGCGTTCCCCAAAA | *rsmZ* overexpression |
| TerF | TGGCGGCCGCTCTAGGAGCTCTACAAATAAGGATCCAAAC | for constructing pBB-*lacZ* |
| TerR | TAGAGAGCGTTCACCGACAAA | for constructing pBB-’*lacZ* |
| Lac-X-F2 | GTCGGTGAACGCTCTCTATGGGTTTCGGGGCCTAGG | for constructing pBB-*rsmX*’-’*lacZ* |
| Lac-X-R2 | AATGAAGATTAAGCCTGTCGCC | for constructing pBB-*rsmX*’-’*lacZ* |
| 6522-X-F3 | ACAGGCTTAATCTTCATTCTGCAGAATTGTGAGCGGATAA | for constructing pBB-*rsmX*’-’*lacZ* |
| Lac-Y-F2 | GTCGGTGAACGCTCTCTAGTACATGGGCGTCATGTAAGC | for constructing pBB-*rsmY*’-’*lacZ* |
| Lac-Y-R2 | GTGAAGTAGATTAGCTCTGGATCC | for constructing pBB-*rsmY*’-’*lacZ* |
| 6522-Y-F3 | AGAGCTAATCTACTTCACCTGCAGAATTGTGAGCGGATAA | for constructing pBB-*rsmY*’-’*lacZ* |
| Lac-Z-F2 | GTCGGTGAACGCTCTCTACGGTACGAGCAATCCTGCCGT | for constructing pBB-*rsmZ*’-’*lacZ* |
| Lac-Z-R2 | GGGCTGATATTAGAGAGTTTCCAT | for constructing pBB-*rsmZ*’-’*lacZ* |
| 6522-Z-F3 | ACTCTCTAATATCAGCCCCTGCAGAATTGTGAGCGGATAA | for constructing pBB-*rsmZ*’-’*lacZ* |
| 6522R | CGGTATCGATAAGCTGAAACGTCTTGCTCGAGATCAA | for constructing pBB-’lacZ |
| Strains | **Genotype and relevant characteristics** | **Reference** |
| HT66 | *P. chlororaphis* Wild-type, PCN, Apr Spr | This study |
| HT66-FLUO | A phenotypic variant of HT66 without PCN biosynthesis and high Pyoverdines production, Apr Spr | This study |
| S17-1(λpir) | E. coli res- pro mod*+ integrated copy of RP4, mob+, used for incorporating constructs into P.chlororaphis* | Lab stock |
| Plasmids |  |  |
| pBBR1MCS | Lac and T7 expression vector, Kmr | Lab stock |
| pBbB5k-GFP | LacUV5 expression vector, Kmr | Lab stock |
| pME6522 | pVS1-p15A shuttle vector for constructing the transcriptional *lacZ* fusions, Tcr | Lab stock |
| pBB-*rsmX*’-’*lacZ* | pBBR1MCS containing a rrnBT1 terminator from pBbB5k-GFP, a 109bp fragment covering from promoter/operator region of *rsmX* gene cluster and *lacZ* gene from pME6522, Kmr | This study |
| pBB-*rsmY*’-’*lacZ* | pBBR1MCS containing a rrnBT1 terminator from pBbB5k-GFP, a 89 bp fragment covering from promoter/operator region of *rsmY* gene cluster and *lacZ* gene from pME6522, Kmr | This study |
| pBB-*rsmZ*’-’*lacZ* | pBBR1MCS containing a rrnBT1 terminator from pBbB5k-GFP, a 301bp fragment covering from promoter/operator region of *rsmZ* gene cluster and *lacZ* gene from pME6522, Kmr | This study |
| pK18mobsacB | Broad-host-range gene replacement vector; sacB, Kmr | Lab stock |

**Table S2:** **Mean transcript abundance and ratio of abundances (Fold change: HT66-FLUO/HT66) of the phenazine genes and related regulating factors in the phenotypic variant compared to the WT strain (p-value < 0.05)**

| **Gene ID** | **Gene** | **Protein description** | **Mean RPKM**  **HT66** | **Mean RPKM**  **HT66-FLUO** | **Fold Change** |
| --- | --- | --- | --- | --- | --- |
| M217_RS0132000 | *gacA* | Response regulator | 403.65 | 266.19 | -1.52 |
| M217_RS0109720 | *gacS* | Sensor protein | 60.53 | 73.82 | 1.22 |
|  |  |  |  |  |  |
| M217_RS0125395 | *rpeB* | transcriptional regulator | 216.31 | 78.55 | -2.75 |
| M217_RS0125400 | *rpeA* | histidine kinase | 123.77 | 37.09 | -3.34 |
| M217_RS0115090 | *rpoS* | Sigma factor | 6541.75 | 2104.67 | -3.11 |
| M217_RS0120615 | *pip* | Phenazine-inducing protein | 147.90 | 104.32 | -1.42 |
| M217_RS0101675 | *psrA* | transcriptional regulator | 236.99 | 128.16 | -1.85 |
| M217_RS0101235 | *rsmE* | Translational regulator | 99.83 | 99.05 | -1.01 |
| M217_RS0109975 | *rsmA* | Translational regulator | 989.99 | 737.80 | -1.34 |
| M217_RS0101875 | *anr* | transcriptional regulator | 360.28 | 946.37 | 2.63 |
| M217_RS0122025 | *retS* | sensor histidine kinase | 138.01 | 49.53 | -2.79 |
| M217_RS0120795 | *ladS* | sensor histidine kinase | 29.29 | 119.22 | 4.07 |
| M217_RS0122480 | *hfq* | RNA chaperone | 1540.28 | 1445.22 | -1.07 |
| M217_RS0107335 | *iopA* | inducer of phenazine A | 1709.14 | 294.99 | -5.79 |
| M217_RS0107345 | *iopB* | inducer of phenazine B | 1177.39 | 228.29 | -5.16 |
|  |  |  |  |  |  |
| M217_RS0112870 | *phzI* | phenazine biosynthesis protein | 284.35 | 39.20 | -7.25 |
| M217_RS0112875 | *phzR* | phenazine biosynthesis protein | 348.02 | 170.44 | -2.04 |
| M217_RS0112880 | *phzA* | phenazine biosynthesis protein | 920.71 | 29.66 | -31.04 |
| M217_RS0112885 | *phzB* | phenazine biosynthesis protein | 1037.94 | 20.19 | -51.40 |
| M217_RS0112890 | *phzC* | phenazine biosynthesis protein | 779.89 | 16.98 | -45.93 |
| M217_RS0112895 | *phzD* | phenazine biosynthesis protein | 608.87 | 7.92 | -76.83 |
| M217_RS0112900 | *phzE* | phenazine biosynthesis protein | 692.98 | 17.80 | -38.93 |
| M217_RS0112905 | *phzF* | phenazine biosynthesis protein | 653.42 | 13.66 | -47.83 |
| M217_RS0112910 | *phzG* | phenazine biosynthesis protein | 583.00 | 11.16 | -52.25 |
| M217_RS0112915 | *phzH* | phenazine biosynthesis protein | 1032.94 | 32.13 | -32.15 |

**Table S3 Mean transcript abundance and ratio of abundances (Fold change HT66-FLUO/HT66) of the genes involve in siderophore in the phenotypic variant compared to the WT strain (p-value < 0.05)**

| **Gene ID** | **Gene** | **Protein description** | **Mean RPKM HT66** | **Mean RPKM HT66-FLUO** | **Fold change** |
| --- | --- | --- | --- | --- | --- |
| **Pyoverdine biosynthesis** | |  |  |  |  |
| M217_RS0105795 | *pvdA* | ornithine monooxygenase | 327.85 | 3315.02 | 10.11 |
| M217_RS0105805 | *pvdR* | hemolysin secretion protein D | 14.82 | 80.30 | 5.42 |
| M217_RS0105810 | *pvdT* | macrolide ABC transporter ATP-binding protein | 11.28 | 44.10 | 3.91 |
| M217_RS0105820 | *pvdP* | RND transporter | 45.78 | 254.43 | 5.56 |
| M217_RS0105825 | *pvdM* | pyoverdin biosynthesis | 40.23 | 128.15 | 3.19 |
| M217_RS0105835 | *pvdO* | pyoverdine responsive serine | 32.96 | 150.09 | 4.55 |
| M217_RS0105840 | *pvdF* | N(5)-hydroxyornithinetransformylase | 134.81 | 1523.52 | 11.30 |
| M217_RS0105845 | *pvdE* | cyclic peptide transporter | 92.40 | 281.12 | 3.04 |
| M217_RS0105855 | *pvdD* | Pyoverdine sidechain non-ribosomal peptide synthetase | 67.86 | 809.80 | 11.93 |
| M217_RS0108350 | *pvdL* | peptide synthase | 99.19 | 895.61 | 9.03 |
| M217_RS0108355 | *pvdS* | RNA polymerase sigma70 | 180.81 | 601.52 | 3.33 |
| M217_RS0108360 | *pvdY* | pyoverdine biosynthesis protein | 74.74 | 544.98 | 7.29 |
| M217_RS0120010 | *pvdH* | peptide synthetase | 38.10 | 413.18 | 10.84 |
| M217_RS0108290 | *pvdJ* | Pyoverdine sidechain non-ribosomal peptide synthetase | 117.16 | 1499.20 | 12.80 |
| M217_RS0127645 | *pvdQ* | Acyl-homoserine lactone acylase | 53.42 | 332.19 | 6.22 |
| **Achromobactin biosynthesis** | |  |  |  |  |
| M217_RS0126125 | *acsS* | RNA polymerase sigma factor | 188.94 | 588.18 | 3.11 |
| M217_RS0126130 |  | sugar ABC transporter substrate-binding protein | 107.86 | 366.73 | 3.40 |
| M217_RS0126135 |  | TonB-dependent ferric achromobactin receptor protein | 25.08 | 2882.14 | 114.90 |
| M217_RS0126140 | *acsF* | Achromobactin biosynthesis protein | 33.46 | 4300.04 | 128.50 |
| M217_RS0126145 | *acsD* | Achromobactin biosynthesis protein | 21.28 | 3257.85 | 153.07 |
| M217_RS0126150 | *acsE* | Achromobactin biosynthesis protein | 13.50 | 2425.19 | 179.60 |
| M217_RS0126155 | *yhcA* | Permease of the major facilitator superfamily | 10.98 | 1530.50 | 139.45 |
| M217_RS0126160 | *acsC* | Uncharacterized siderophore S biosynthesis protein | 33.40 | 2667.25 | 79.86 |
| M217_RS0126165 | *acsB* | Achromobactin biosynthesis protein AcsB | 9.56 | 1376.33 | 143.90 |
| M217_RS0126170 | *acsA* | Achromobactin biosynthesis protein AcsA | 16.75 | 2899.20 | 173.10 |
| M217_RS0126175 | *cbrA* | Siderophoreachromobactin ABC transporter | 11.74 | 1760.19 | 149.94 |
| M217_RS0126180 | *cbrB* | Siderophoreachromobactin ABC transporter | 8.32 | 695.40 | 83.57 |
| M217_RS0126185 | *cbrC* | Siderophoreachromobactin ABC transporter | 7.19 | 540.28 | 75.13 |
| M217_RS0126190 | *cbrD* | Siderophoreachromobactin ABC transporter | 11.32 | 867.68 | 76.68 |
| M217_RS0126195 |  | Uncharacterized Fe-S protein | 15.80 | 703.81 | 44.55 |
| M217_RS0126200 |  | Putative achromobactin biosynthesis protein | 15.20 | 873.21 | 57.45 |
| **Haem acquisition** |  |  |  |  |  |
| M217_RS0120560 | *hasF* | outer membrane protein HasF | 4.62 | 21.42 | 4.63 |
| M217_RS0120565 | *hasE* | hemolysin D | 1.85 | 33.16 | 17.91 |
| M217_RS0120570 | *hasD* | peptidase | 2.26 | 25.18 | 11.14 |
| M217_RS0120575 | *hasAP* | heme acquisition protein HasAp | 71.13 | 4489.07 | 63.11 |
| M217_RS0120580 | *hasR* | ligand-gated channel | 13.03 | 129.80 | 9.96 |
| M217_RS0120585 | *hasS* | siderophore-interacting protein | 34.72 | 108.44 | 3.12 |
| M217_RS0120590 | *hasI* | RNA polymerase sigma70 | 19.79 | 70.61 | 3.57 |
| M217_RS0110610 | *phuR* | TonB-dependent outermembraneheme receptor | 77.75 | 275.00 | 3.54 |
| M217_RS0110615 | *hemO* | heme oxygenase | 243.91 | 382.85 | 1.57 |
| M217_RS0107925 | *phuW* | lipoprotein | 49.25 | 197.83 | 4.02 |
| M217_RS0107930 | *phuV* | hemin importer ATP-binding subunit | 62.90 | 107.33 | 1.71 |
| M217_RS0107935 | *phuU* | hemin ABC transporter permease | 54.04 | 83.22 | 1.54 |
| M217_RS0107940 | *phuT* | hemin ABC transporter periplasmic hemin-binding protein | 115.07 | 223.77 | 1.94 |

**Table S4 Mean transcript abundance and ratio of abundances (Fold change HT66-FLUO/HT66) of the genes involve in secretion system in the phenotypic variant compared to the WT strain (*p* value < 0.05)**

| **Gene ID** | **Gene** | **Protein description** | **Mean RPKM HT66** | **Mean RPKM HT66-FLUO** | **Fold change** |
| --- | --- | --- | --- | --- | --- |
| **H1-T6SS** |  |  |  |  |  |
| M217_RS0132395 |  | hypothetical protein | 183.57 | 9.67 | -18.99 |
| M217_RS0132400 |  | 3-oxoacyl-ACP synthase | 89.41 | 6.68 | -13.39 |
| M217_RS0132405 |  | hypothetical protein | 130.43 | 3.68 | -35.42 |
| M217_RS0132410 |  | hypothetical protein | 208.61 | 6.25 | -33.39 |
| M217_RS0132415 | *vgrG* | type VI secretion protein ImpA | 188.92 | 6.09 | -31.04 |
| M217_RS0132420 | *clpV1* | ATPase AAA | 260.55 | 5.31 | -49.05 |
| M217_RS0132425 | *hisH1* | type VI secretion protein | 228.82 | 7.40 | -30.93 |
| M217_RS0132430 | *hsiG1* | hypothetical protein | 149.05 | 3.94 | -37.80 |
| M217_RS0132435 | *hsiF1* | hypothetical protein | 217.29 | 3.90 | -55.70 |
| M217_RS0132445 |  | hypothetical protein | 688.43 | 5.19 | -132.52 |
| M217_RS0132450 | *hsiE1* | Hypothetical protein | 924.46 | 5.84 | -158.20 |
| M217_RS0132455 | *hcp1* | hypothetical protein | 3371.00 | 25.20 | -133.78 |
| M217_RS0132460 | *hsiC1* | EvpB family type VI secretion protein | 1599.80 | 11.47 | -139.53 |
| M217_RS0132465 | *hsiB1* | hypothetical protein | 1372.66 | 9.79 | -140.17 |
| M217_RS0132470 | *hsiA1* | type VI secretion protein ImpA | 408.10 | 5.88 | -69.46 |
| M217_RS0132475 | *fha1* | signal peptide protein | 419.34 | 38.73 | -10.83 |
| M217_RS0132480 | *lip1* | type VI secretion protein | 379.58 | 6.79 | -55.90 |
| M217_RS0132485 | *hisJ1* | type VI secretion protein | 311.70 | 12.39 | -25.16 |
| M217_RS0132490 | *dotU1* | hypothetical protein | 253.72 | 9.95 | -25.51 |
| M217_RS0132495 | *icmF1* | type VI secretion protein IcmF | 219.31 | 8.40 | -26.11 |
| M217_RS0132500 | *pppB* | type VI secretion system protein ImpM | 228.46 | 7.84 | 29.15 |
| M217_RS0132505 | *pppA* | PppA | 183.17 | 7.20 | -25.44 |
| M217_RS0132510 | *ppkA* | serine/threonine protein kinase | 184.15 | 7.97 | -23.10 |
| M217_RS0132515 | *taqT1* | ABC transporter ATP-binding protein | 138.21 | 6.90 | -20.04 |
| M217_RS0132520 | *taqS1* | membrane protein | 78.71 | 3.47 | -22.69 |
| M217_RS0132525 | *taqR1* | type VI secretion protein | 57.61 | 5.67 | -10.16 |
| M217_RS0132530 | *taqQ1* | hypothetical protein | 895.94 | 63.99 | -14.00 |
| **H2-T6SS** |  |  |  |  |  |
| M217_RS0117290 | *hsiA2* | type VI secretion protein | 80.72 | 7.64 | -10.57 |
| M217_RS0117295 | *hisB2* | hypothetical protein | 681.93 | 17.31 | -39.40 |
| M217_RS0117300 | *hsiC2* | type VI secretion protein | 592.59 | 18.21 | -32.54 |
| M217_RS0117305 | *hsiF2* | hypothetical protein | 111.72 | 4.77 | -23.41 |
| M217_RS0117325 |  | hypothetical protein | 34.71 | 7.82 | -4.44 |
| M217_RS0117330 |  | hypothetical protein | 34.87 | 7.16 | -4.87 |
| M217_RS0117335 | *hsiG2* | type VI secretion protein | 59.37 | 10.60 | -5.60 |
| M217_RS0117340 | *hsiH2* | type VI secretion protein | 51.35 | 5.31 | -9.68 |
| M217_RS0117345 | *clpV2* | ATPase AAA | 101.35 | 8.65 | -11.72 |
| M217_RS0117350 | *sfa2* | Fis family transcriptional regulator | 64.39 | 7.28 | -8.85 |
| M217_RS0117360 | *fha2* | signal peptide protein | 94.50 | 7.56 | -12.49 |
| M217_RS0117365 | *lip2* | type VI secretion protein | 68.44 | 8.98 | -7.62 |
| M217_RS0117370 | *hsiJ2* | type VI secretion protein | 89.05 | 11.31 | -7.87 |
| M217_RS0117375 | *dotU2* | membrane protein | 98.26 | 6.89 | -14.26 |
| M217_RS0117380 | *icmF2* | type VI secretion protein VasK | 97.22 | 7.93 | -12.26 |
| M217_RS0117385 | *stp1* | protein phosphatase | 77.56 | 8.64 | -8.98 |
| M217_RS0117390 | *stk1* | serine/threonine protein kinase | 27.66 | 6.12 | -4.52 |
| M217_RS0124340 | *hcp2* | type VI secretion system secreted protein | 2937.28 | 74.19 | -39.59 |
| M217_RS0124345 | *vgrG2* | type VI secretion system secreted protein | 56.32 | 6.45 | -8.73 |
| **H3-T6SS** |  |  |  |  |  |
| M217_RS0131025 | *vgrG* | type VI secretion protein | 181.62 | 70.63 | -2.57 |
| **Hxc-T2SS** |  |  |  |  |  |
| M217_RS0127975 | *hxcS* | type II secretion protein | 54.32 | 108.90 | 2.00 |
| M217_RS0127980 | *hxcR* | type II secretion protein | 60.59 | 106.86 | 1.76 |
| M217_RS0127990 | *hxcO* | type II secretion protein | 112.08 | 166.09 | 1.48 |
| M217_RS0127995 | *hxcZ* | type II secretion protein | 90.79 | 140.76 | 1.55 |
| M217_RS0128000 | *hxcY* | type II secretion protein | 110.43 | 242.23 | 2.19 |
| M217_RS0128005 | *hxcX* | type II secretion protein | 173.87 | 293.78 | 1.69 |
| M217_RS0128010 | *hxcT* | type II secretion protein | 293.52 | 476.27 | 1.62 |
| M217_RS0128015 | *hxcV* | type II secretion protein | 98.84 | 187.80 | 1.90 |
| M217_RS0128020 | *hxcP* | type II secretion protein | 217.94 | 270.08 | 1.24 |
| M217_RS0128025 | *hxcU* | type II secretion protein | 46.34 | 108.14 | 2.33 |
| M217_RS0128030 | *hxcW* | type II secretion protein | 32.46 | 77.13 | 2.38 |
| M217_RS0128035 | *vreR* | type II secretion protein | 142.77 | 1109.02 | 7.77 |
| M217_RS0128040 | *vreI* | type II secretion protein | 109.85 | 147.90 | 1.35 |
| M217_RS0128045 | *vreA* | type II secretion protein | 318.26 | 384.66 | 1.21 |

**Table S5 Mean transcript abundance and ratio of abundances (Fold change HT66-FLUO/HT66) of the genes involve in Nitrogen metabolism and phosphate metabolism of the phenotypic variant compared to the WT strain (p value < 0.05)**

| **Gene ID** | **Gene** | **Protein description** | **Mean RPKM HT66** | **Mean RPKM HT66-FLUO** | **Fold Change** | |
| --- | --- | --- | --- | --- | --- | --- |
| **Nitrate uptake and metabolism(Nitrogen metabolism)** | | |  |  | |  |
| M217_RS0102040 | *nasR* | nitrite reductase | 2.83 | 213.32 | | 75.48 |
| M217_RS0102045 | *nirD* | nitrite reductase | 1.20 | 92.48 | | 76.90 |
| M217_RS0102050 | *nasA* | nitrate reductase | 4.27 | 160.37 | | 37.51 |
| M217_RS0102020 | *ntrA* | nitrate transporter | 10.44 | 930.08 | | 89.12 |
| M217_RS0117720 | *glnK* | Nitrogen regulatory protein PII | 862.02 | 12545.64 | | 14.55 |
| M217_RS0117725 | *amtB* | Ammonia permease | 118.04 | 7930.64 | | 67.19 |
| M217_RS0122130 | *urtA* | branched-chain amino acid ABC transporter substrate-binding protein | 35.67 | 7960.66 | | 223.19 |
| M217_RS0122135 | *urtB* | urea ABC transporter permease | 10.01 | 1293.04 | | 129.18 |
| M217_RS0122140 | *urtC* | amino acid ABC transporter permease | 6.63 | 1161.25 | | 175.18 |
| M217_RS0122145 | *urtD* | urea ABC transporter ATP-binding protein | 8.97 | 1944.14 | | 216.71 |
| M217_RS0122150 | *urtE* | urea ABC transporter ATP-binding protein | 13.02 | 2208.16 | | 169.57 |
| M217_RS0122155 | *ureD* | urease accessory protein UreD | 40.12 | 1023.32 | | 25.51 |
| M217_RS0122160 | *ureA* | urease subunit gamma | 48.95 | 682.93 | | 13.95 |
| M217_RS0122165 |  | acetyltransferase | 49.89 | 878.41 | | 17.61 |
| M217_RS0122170 | *ttr* | acetyltransferase | 61.56 | 893.50 | | 14.52 |
| M217_RS0122175 | *ureB* | urease subunit beta | 44.43 | 711.66 | | 16.02 |
| M217_RS0122180 | *ureC* | urease subunit alpha | 54.01 | 793.70 | | 14.70 |
| M217_RS0122260 | *ureE* | urease accessory protein UreE | 12.52 | 2051.76 | | 163.86 |
| M217_RS0122265 | *ureF* | urease accessory protein UreF | 15.86 | 1433.81 | | 90.39 |
| M217_RS0122270 | *ureG* | urease accessory protein UreG | 16.50 | 2264.71 | | 137.23 |
| M217_RS0122275 | *ureJ* | protein hupE | 17.76 | 2462.25 | | 138.61 |
| M217_RS0122280 | *ureG* | N-acylglucosamine 2-epimerase | 36.60 | 110.75 | | 3.03 |
| M217_RS0123320 | *glnA* | glutamine synthetase | 800.79 | 15385.85 | | 19.21 |
| M217_RS0123380 | *ntrB* | two-component system,nitrogen regulation sensor histidine kinase | 32.73 | 1402.25 | | 42.84 |
| M217_RS0123385 | *ntrC* | two-component system,nitrogen regulation response regulator | 61.88 | 3586.80 | | 57.96 |
| **Phosphate transport** | |  |  |  | |  |
| M217_RS0117205 | *pstS* | phosphate-binding protein | 14871.58 | 2606.45 | | -5.71 |
| M217_RS0117210 | *pstC* | phosphate ABC transporter permease | 503.22 | 131.91 | | -3.81 |
| M217_RS0117215 | *pstA* | phosphate ABC transporter permease | 690.80 | 205.07 | | -3.37 |
| M217_RS0117220 | *pstB* | phosphate ABC transporter ATP-binding protein | 902.33 | 214.54 | | -4.21 |
| M217_RS0117225 | *phoU* | Phosphate transport system regulatory protein | 857.82 | 188.62 | | -4.55 |
| M217_RS0117250 | *PhoR* | two-component system,phosphate regulon sensor histidine kinase | 219.78 | 60.27 | | -3.65 |
| M217_RS0117255 | *PhoB* | two-component system, phosphate regulon response regulator | 596.72 | 116.12 | | -5.14 |

**Table S6 Mean transcript abundance and ratio of abundances (Fold change HT66-FLUO/HT66) of the genes involve in flagellar and pilus genes of the phenotypic variant compared to the WT strain (p value < 0.05)**

| **Gene ID** | **Gene** | **Protein description** | **Mean RPKM HT66** | **Mean RPKM HT66-FLUO** | **Fold Change** | |
| --- | --- | --- | --- | --- | --- | --- |
| **Flagellar genes** |  |  |  |  | |  |
| M217_RS0107045 | *fliH* | flagellar assembly protein FliH | 49.28 | 124.41 | | 2.52 |
| M217_RS0107050 | *fliI* | ATP synthase | 38.03 | 103.72 | | 2.73 |
| M217_RS0107055 | *fliJ* | flagellar biogenesis protein | 23.97 | 61.67 | | 2.57 |
| M217_RS0107120 | *flhA* | flagellar biosynthesis protein FlhA | 88.05 | 214.28 | | 2.43 |
| **Pili genes** |  |  |  |  | |  |
| M217_RS0116520 | *pilZ* | pilus assembly protein PilZ | 39.97 | 327.16 | | 8.19 |
| M217_RS0120065 | *pilA* | fimbrial protein | 14.40 | 53.84 | | 3.74 |
| M217_RS0121845 | *flp* | pilus assembly protein | 88.11 | 370.96 | | 4.21 |
| M217_RS0121850 | *cpaB* | pilus assembly protein CpaB | 107.37 | 347.64 | | 3.24 |
| M217_RS0121860 | *tadZ* | pilus assembly protein | 13.60 | 126.87 | | 9.33 |
| M217_RS0121865 | *tadA* | ATPase | 7.97 | 116.73 | | 14.64 |
| M217_RS0121870 | *tadB* | type II secretion system protein F | 5.67 | 87.65 | | 15.45 |
| M217_RS0121875 | *tadC* | type II secretion system protein F | 9.52 | 94.45 | | 9.92 |
| M217_RS0121890 | *tadE* | pilus assembly protein TadE | 44.71 | 141.73 | | 3.17 |
|  |  |  |  |  | |  |
| M217_RS0106160 | *cupB1* | fimbrial protein | 45.19 | 5.81 | | -7.77 |
| M217_RS0110735 | *cpaF* | pilus assembly protein CpaF | 132.80 | 60.44 | | -2.20 |
| M217_RS0110740 |  | chemotaxis protein CheY | 149.28 | 70.57 | | -2.12 |
| M217_RS0110745 |  | membrane protein | 79.80 | 30.46 | | -2.62 |
| M217_RS0110750 |  | pilus assembly protein TadG | 69.85 | 21.34 | | -3.27 |
| M217_RS0110755 |  | pilus assembly protein | 111.59 | 25.98 | | -4.30 |
| M217_RS0110760 | *cpaB* | pilus assembly protein CpaB | 184.07 | 19.07 | | -9.65 |
| M217_RS0110765 |  | ATPase AAA | 200.22 | 18.29 | | -10.95 |
| M217_RS0110770 |  | membrane protein | 820.54 | 26.51 | | -30.95 |
| M217_RS0110775 | *pilA* | pilus assembly protein PilA | 1148.08 | 44.66 | | -25.71 |
| M217_RS0110780 | *pilA* | pilus assembly protein PilA | 1206.64 | 330.46 | | -3.65 |
| M217_RS0121560 |  | molecular chaperone | 40.00 | 13.51 | | -2.96 |
| M217_RS0124345 |  | type IV secretion protein Rhs | 56.32 | 6.45 | | -8.73 |
| M217_RS0124350 |  | hypothetical protein | 32.99 | 10.20 | | -3.23 |
| M217_RS0124355 |  | type IV secretion protein Rhs | 66.84 | 20.18 | | -3.31 |
| M217_RS0132590 |  | pilus assembly protein | 46.18 | 19.19 | | -2.41 |
| M217_RS0132595 |  | fimbrial protein | 376.46 | 27.62 | | -13.63 |

**Table S7 List of SNP in *P. chlororaphis* HT66-FLUO in whole genome resequencing**

| ref | ref_base<->  sample_base | ref_aa<->  sample_aa | mutate_  type | ref_gene_ID | Locus Tag |  |
| --- | --- | --- | --- | --- | --- | --- |
| contig000011_11078 | G<->C | G<->G | syn | CDS5917322 | M217_RS0102875 | |
| contig000011_11079 | C<->T | L<->L | syn | CDS5917322 | M217_RS0102875 | |
| contig000011_11082 | T<->C | L<->L | syn | CDS5917322 | M217_RS0102875 | |
| contig000011_11085 | G<->C | G<->C | nonsyn | CDS5917322 | M217_RS0102875 | |
| contig000011_11090 | T<->C | G<->G | syn | CDS5917322 | M217_RS0102875 | |
| contig000011_11121 | A<->C | S<->R | nonsyn | CDS5917322 | M217_RS0102875 | |
| contig000011_11122 | G<->C | S<->T | nonsyn | CDS5917322 | M217_RS0102875 | |
| contig000011_11126 | G<->C | L<->L | syn | CDS5917322 | M217_RS0102875 | |
| contig000011_11138 | T<->C | R<->R | syn | CDS5917322 | M217_RS0102875 | |
| contig000011_11153 | G<->A | L<->L | syn | CDS5917322 | M217_RS0102875 | |
| contig000011_11162 | C<->G | L<->L | syn | CDS5917322 | M217_RS0102875 | |
| contig000011_11169 | A<->C | S<->R | nonsyn | CDS5917322 | M217_RS0102875 | |
| contig000011_11173 | G<->A | R<->H | nonsyn | CDS5917322 | M217_RS0102875 | |
| contig000011_11178 | C<->G | Q<->E | nonsyn | CDS5917322 | M217_RS0102875 | |
| contig000011_11180 | G<->A | Q<->Q | syn | CDS5917322 | M217_RS0102875 | |
| contig000011_11182 | C<->T | A<->V | nonsyn | CDS5917322 | M217_RS0102875 | |
| contig000011_11183 | A<->G | A<->A | syn | CDS5917322 | M217_RS0102875 | |
| contig000011_11198 | C<->T | N<->N | syn | CDS5917322 | M217_RS0102875 | |
| contig000011_11207 | G<->C | V<->V | syn | CDS5917322 | M217_RS0102875 | |
| contig000011_11209 | T<->C | V<->A | nonsyn | CDS5917322 | M217_RS0102875 | |
| contig000011_11219 | C<->T | R<->R | syn | CDS5917322 | M217_RS0102875 | |
| contig000011_11225 | G<->A | Q<->Q | syn | CDS5917322 | M217_RS0102875 | |
| contig000011_11227 | A<->T | Q<->L | nonsyn | CDS5917322 | M217_RS0102875 | |
| contig000011_11234 | T<->G | G<->G | syn | CDS5917322 | M217_RS0102875 | |
| contig000011_11238 | C<->G | L<->V | nonsyn | CDS5917322 | M217_RS0102875 | |
| contig000011_11243 | G<->A | V<->V | syn | CDS5917322 | M217_RS0102875 | |
| contig000011_11245 | A<->T | E<->V | nonsyn | CDS5917322 | M217_RS0102875 | |
| contig000011_11248 | T<->A | L<->Q | nonsyn | CDS5917322 | M217_RS0102875 | |
| contig000011_11255 | G<->C | P<->P | syn | CDS5917322 | M217_RS0102875 | |
| contig000018_9858 | T<->C | H<->H | syn | CDS5919997 | M217_RS0107785 | |
| contig000018_9873 | T<->C | S<->S | syn | CDS5919997 | M217_RS0107785 | |
| contig000049_44180 | T<->G | F<->V | nonsyn | CDS5930871 | M217_RS0131460 | |
| contig000049_44188 | G<->A | L<->L | syn | CDS5930871 | M217_RS0131460 | |
| contig000049_44193 | T<->A | V<->D | nonsyn | CDS5930871 | M217_RS0131460 | |

**Table S8 List of InDel in *P. chlororaphis* HT66-FLUO in whole genome resequencing**

|  |  | |  | Mutate type | base | CDS with  InDel | ref_gene_ID | Locus Tag |
| --- | --- | --- | --- | --- | --- | --- | --- | --- |
| contig000002 | 42550 | 42550 | | Insertion | A | no | CDS5915893 | M217_RS0100230～M217_RS0100235 |
| contig000003 | 345 | 345 | | Insertion | G | yes | CDS5915936 | M217_RS33245 |
| contig000018 | 4865 | 4865 | | Insertion | T | yes | CDS5919990 | M217_RS33450 |
| contig000023 | 249626 | 249626 | | Insertion | A | no | CDS5921282 | M217_RS0110565～M217_RS0110570 |
| contig000025 | 57731 | 57731 | | Insertion | T | no | CDS5922936 | M217_RS0113305～M217_RS0113310 |
| contig000028 | 464913 | 464913 | | Insertion | T | yes | CDS5925342 | M217_RS0118225 |
| contig000028 | 464912 | 464913 | | Deletion | C | yes | CDS5925343 | M217_RS0118225 |
| contig000038 | 12658 | 12659 | | Deletion | A | no | CDS15179091 | M217_RS0121545～M217_RS0121550 |
| contig000049 | 40629 | 40629 | | Insertion | TA | no | CDS5930867 | M217_RS0131450～M217_RS0131455 |
| contig000049 | 139699 | 139700 | | Deletion | G | no | CDS5931039 | M217_RS0131880～M217_RS0131895 |
| contig000050 | 149867 | 149867 | | Insertion | GCC | yes | CDS11934795 | M217_RS0132620 |
